# A novel thiazole-sulfonamide hybrid molecule as a promising dual tubulin/carbonic anhydrase IX inhibitor with anticancer activity

**DOI:** 10.3389/fchem.2025.1606848

**Published:** 2025-06-26

**Authors:** Hussam Elddin Nabeih Khasawneh, Elryah I. Ali, Ranya Mohammed Elmagzoub, Raed Fanoukh Aboqader Al-Aouadi, Wesam Taher Almagharbeh, Ghallab Alotaibi, Stefan Bräse, Abdullah Alkhammash

**Affiliations:** ^1^ Chemical Engineering Department, Al-Huson University College, Al-Balqa’ Applied University, Al-Salt, Jordan; ^2^ Department of Medical Laboratory Technology, College of Applied Medical Sciences, Northern Border University, Arar, Saudi Arabia; ^3^ College of Medicine, Al-Ayen Iraqi University, AUIQ, An Nasiriyah, Iraq; ^4^ Medical and Surgical Nursing Department, Faculty of Nursing, University of Tabuk, Tabuk, Saudi Arabia; ^5^ Department of Pharmacology, College of Pharmacy, Al-Dawadmi Campus, Shaqra University, Shaqra, Saudi Arabia; ^6^ Institute of Biological and Chemical Systems, Functional Molecular Systems (IBCS-FMS), Karlsruhe Institute of Technology (KIT), Karlsruhe, Germany

**Keywords:** thiazole, chalcone, sulphonamide, tubulin, carbonic anhydrase, cytotoxicity, apoptosis

## Abstract

**Introduction:**

Multitargeted anticancer agents can overcome resistance by simultaneously modulating key pathways. This study reports a novel thiazole -chalcone/sulfonamide hybrid (compound 7) designed to inhibit both tubulin polymerization and carbonic anhydrase IX (CA IX).

**Methods:**

Compound 7 was synthesized through a five-step sequence and characterized by NMR and elemental analysis. Its cytotoxicity was assessed against cancer (HT-29, A549, 786-O, MCF-7) and normal (WI-38) cell lines. Tubulin polymerization and CA isoform inhibition (I, II, IX, XII) were evaluated. Apoptosis induction was confirmed by measuring p53, Bax, Bcl-2, and caspases 3 and 9. Molecular docking, ADMET, and DFT studies supported mechanistic insights.

**Results and discussion:**

Compound 7 showed potent activity against HT-29 cells (IC_50_ = 0.98 μM) and low toxicity toward WI-38 cells. It inhibited tubulin polymerization (IC_50_ = 2.72 μM) and selectively targeted CA IX (IC_50_ = 0.021 μM) and CA XII, while sparing CA I and II. Apoptotic effects were confirmed by increased p53 and Bax, reduced Bcl-2, and activation of caspases. Docking studies revealed key interactions within the colchicine-binding site of tubulin and CA IX’s zinc-binding pocket. ADMET and DFT results supported its drug-like properties and electronic suitability. These findings suggest that compound 7 is a promising lead for dual-targeted anticancer therapy with selective cytotoxicity and mechanistic efficacy.

## 1 Introduction

Cancer is a complex and heterogeneous disease involving multiple dysregulated pathways that contribute to uncontrolled proliferation, evasion of apoptosis, angiogenesis, and metastasis (Hanahan and Weinberg, 2011; Vogelstein et al., 2013). Traditional chemotherapeutics often target a single pathway, which can be bypassed by cancer cells, leading to resistance and limited long-term efficacy. Among other advanced theranostic methods (Sgouros et al., 2020; Klika et al., 2021; Makarem et al., 2019), multitarget-directed ligands (MTDLs) have emerged as a promising strategy, offering the ability to modulate multiple cancer-related targets with a single compound (Doostmohammadi et al., 2024; Brindisi et al., 2022). This approach enhances therapeutic efficacy, reduces the likelihood of resistance, and improves treatment outcomes compared to monotherapies or combination regimens. Thus, the rational design of multitarget anticancer agents holds great potential in cancer drug discovery, enabling the simultaneous disruption of multiple tumor-promoting mechanisms and providing a comprehensive approach to address the complexity of tumor biology (Li et al., 2021; Lembo and Bottegoni, 2024).

Among the diverse molecular targets explored in recent years, carbonic anhydrase (CA), particularly the tumor-associated isoforms IX and XII, has garnered significant attention (Supuran, 2018). These membrane-bound enzymes are crucial for regulating pH homeostasis in hypoxic tumor microenvironments by the reversible hydration of carbon dioxide (Mboge et al., 2018). Overexpression of CA IX, driven by hypoxia-inducible factors, is strongly associated with tumor aggressiveness, poor prognosis, and resistance to conventional therapies, as it facilitates extracellular acidification and promotes invasion and metastasis (Chiche et al., 2008). Selective inhibition of CA IX/XII disrupts the acidic pH gradient essential for tumor survival, leading to impaired proliferation and increased sensitivity to other anticancer agents while sparing normal tissues (Neri and Supuran, 2011; Lou et al., 2011).

**FIGURE 1:**
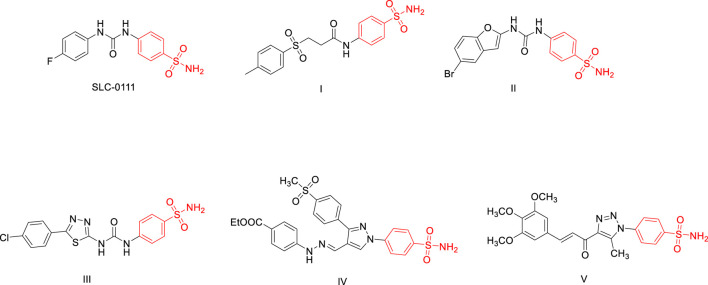
Examples of reported sulphonamides as carbonic anhydrase inhibitors.

Sulfonamides represent the most extensively studied class of CA inhibitors (Carta et al., 2014; Khokhlov et al., 2023), with several derivatives demonstrating promising anticancer activity through selective inhibition of CA IX/XII (McDonald et al., 2022). The ureido-substituted sulfonamide SLC-0111 has advanced to Phase Ib/II clinical trials for metastatic pancreatic cancer, demonstrating the clinical viability of this approach (Kalinin et al., 2021). In recent investigations, the structural optimization of sulfonamide frameworks has led to the development of significantly improved inhibitors. Compound **I** displayed stronger inhibition than SLC-0111 against CA IX and exhibited potent antiproliferative activity against HCT-116 and MCF-7 cells (Elbadawi et al., 2021). Compound **II**, a benzofuran-based sulfonamide, exhibited exceptional CA IX selectivity with a K_i_ of 5.5 nM and favorable selectivity over CA II (Shaldam et al., 2021). The thiadiazole analogue **III** demonstrated a K_i_ of 7.9 nM against CA IX, outperforming SLC-0111 (K_i_ = 45 nM) (Abo-Ashour et al., 2019). Furthermore, compound **IV**, a pyrazole benzenesulfonamide, exhibited potent inhibition of CA XII with an IC_50_ of 0.101 μM and notable anticancer efficacy across several tumor cell lines (Ahmed et al., 2023). Lastly, compound **V**, a 1,2,3-triazole benzenesulfonamide, achieved a K_i_ of 0.03 μM for CA IX and demonstrated dual cytotoxic activity against MCF-7 and Hep-3B cells with high selectivity indices (Abdelhakeem et al., 2024). Examples of such sulfonamide-based inhibitors are illustrated in Figure 1.

In addition to targeting metabolic adaptation, disrupting mitotic progression has long been an effective strategy in cancer treatment (van Vuuren et al., 2015). Among the most validated targets in this context is tubulin, a structural protein essential for the formation of the mitotic spindle and cell division. The dynamic polymerization and depolymerization of microtubules during cell cycle progression render tubulin particularly susceptible to pharmacological intervention (Jordan and Wilson, 2004). Tubulin-binding agents, such as paclitaxel and vinblastine, have demonstrated significant clinical efficacy by inducing mitotic arrest and apoptosis in rapidly dividing cells (Dumontet and Jordan, 2010). However, limitations, including dose-limiting toxicities and the emergence of resistance mechanisms (Kavallaris, 2010)—such as changes in β-tubulin isoforms and increased drug efflux—highlight the need for novel tubulin inhibitors with improved specificity and tolerability.

**FIGURE 2:**
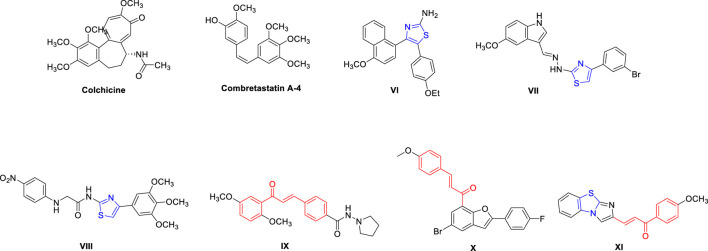
Examples of reported thiazoles and chalcones as inhibitors of tubulin polymerization.

In response to these challenges, various heterocyclic scaffolds have been explored for their ability to disrupt tubulin dynamics (Hawash, 2022; Elshamsy et al., 2023), with thiazole derivatives standing out as particularly promising candidates. Thiazole derivatives, a class of nitrogen- and sulfur-containing heterocycles, have emerged as valuable pharmacophores in anticancer drug discovery (Kaur and Jaitak, 2022) due to their structural rigidity, electronic properties, and ability to engage in specific interactions with biological targets. In particular, their affinity for the colchicine-binding site on tubulin has made them attractive scaffolds for designing inhibitors of tubulin polymerization (Figure 2). The disruption of microtubule dynamics by these agents leads to mitotic arrest and apoptosis in cancer cells. Several potent thiazole-based tubulin inhibitors have been reported. For instance, compound **VI**, a thiazole-naphthalene hybrid, inhibited tubulin polymerization with an IC_50_ of 3.3 μM (Wang et al., 2021). Compound **VII**, a thiazole-hydrazone-indole conjugate, showed even stronger inhibition (IC_50_ = 1.68 μM) (Wang et al., 2024), while compound **VIII**, a 2,4-disubstituted thiazole, exhibited comparable activity (IC_50_ = 2.00 μM) (El-Abd et al., 2022). These examples underscore the potential of thiazole-based molecules as effective antimitotic agents.

Chalcones have also emerged as important scaffolds in the design of multitargeted anticancer agents, particularly for disrupting microtubule dynamics. These α,β-unsaturated carbonyl compounds are well recognized for their ability to interfere with tubulin polymerization by binding to the colchicine site (Liu et al., 2022; Al-Wahaibi et al., 2025), thereby inducing mitotic arrest and apoptosis. Several chalcone-based compounds have demonstrated potent tubulin inhibitory activity (Figure 2). For instance**,** compound **IX**, a 2′,5′-dimethoxychalcone, has been shown to interfere with tubulin polymerization and induce cell cycle arrest in cancer cells (Lin et al., 2011). In another study**,** compound **X**, a benzofuran-chalcone hybrid, demonstrated tubulin-binding potential, as supported by molecular docking analyses (Mphahlele et al., 2018). Another notable example is compound **XI**, a benzo[d]imidazo (Vogelstein et al., 2013) thiazole-chalcone conjugate, which effectively inhibits tubulin assembly and induces apoptosis (Sultana et al., 2018). Collectively, these findings highlight the structural and functional relevance of both thiazoles and chalcones as foundational scaffolds for the development of novel tubulin-targeting anticancer agents.

**FIGURE 3:**
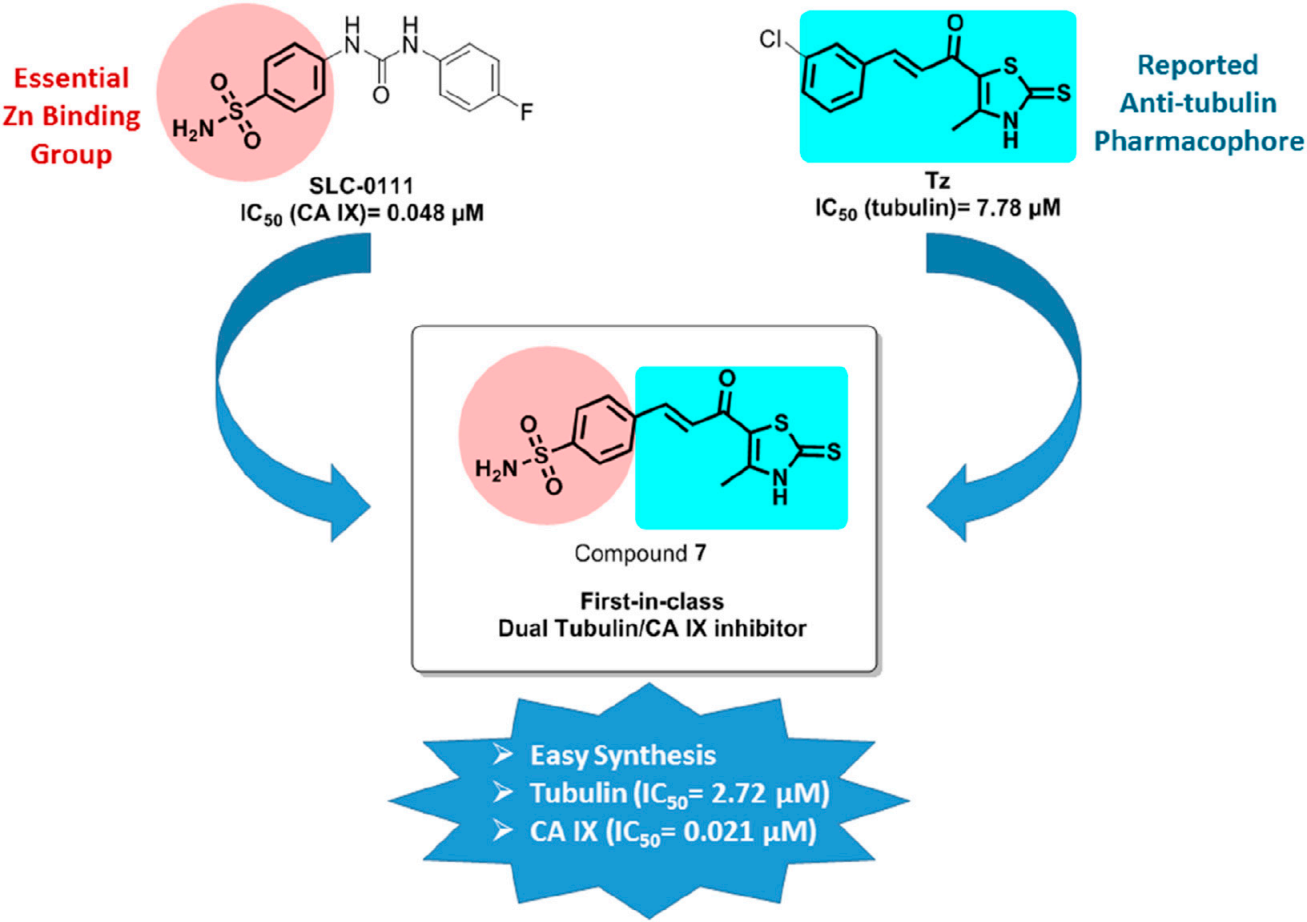
Design of the target compound (compound **7**).

In our earlier investigation aimed at discovering novel antitubulin agents, we identified the lead compound **2e**—hereafter referred to as **Tz**—as a promising thiazole–chalcone derivative with potent inhibitory activity against tubulin polymerization (Hashem et al., 2024). **Tz** demonstrated significant inhibition of tubulin polymerization (IC_50_ = 7.78 μM), although it was slightly less potent than the reference compound Combretastatin A-4. Motivated by the goal of developing a multitargeted anticancer agent, we aimed to enhance the activity of **Tz** by introducing a para-substituted sulfonamide group in place of the original 3-chlorophenyl moiety. This design strategy was inspired by the structural features of the known CA IX inhibitor SLC-0111, where the sulfonamide group coordinates with the active-site zinc ion and engages in hydrogen bonding with polar residues. Recent studies have shown that dual inhibition of tubulin and CA IX/XII within a single molecular framework can produce enhanced anticancer effects through complementary mechanisms (Elkotamy et al., 2025), further supporting our rationale. In our design, the resulting hybrid—compound **7**—retained the thiazole–chalcone pharmacophore essential for inhibiting tubulin polymerization while mimicking both the para-sulfonamide and urea carbonyl groups of SLC-0111 (Figure 3). This structural convergence was intended to allow dual interaction within the colchicine-binding site of tubulin and the active site of CA IX, with the sulfonamide potentially enhancing tubulin binding through additional polar contacts.

To evaluate its biological potential, compound **7** was synthesized and subjected to a comprehensive panel of biological assays, including cytotoxicity screening, tubulin polymerization inhibition, and inhibition of CA isoforms. Additionally, mechanistic studies were carried out to assess its potential to induce apoptosis, followed by a detailed set of molecular modeling studies, including molecular docking to investigate binding interactions, ADMET analysis to predict pharmacokinetic behavior, and DFT calculatio**ns** to examine its electronic properties.

## 2 Results and discussion

### 2.1 Chemistry

**SCHEME 1:**
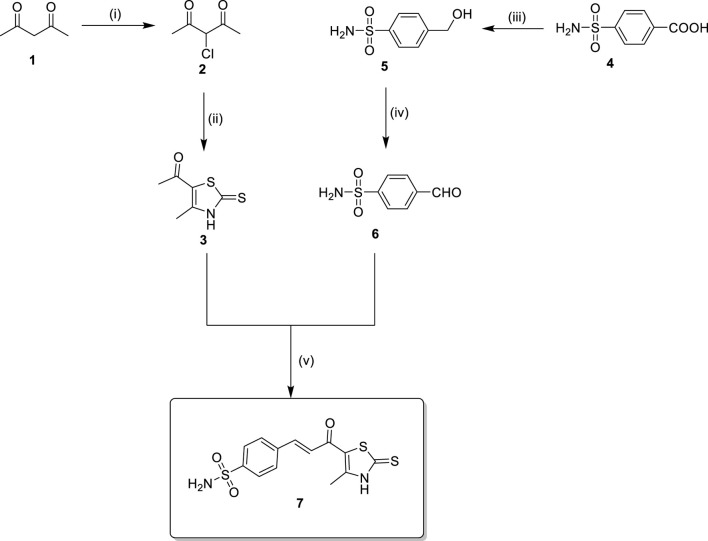
Synthesis of the target compound (**7**).

The synthetic pathway for the chalcone derivative (**7**) is illustrated in Scheme 1 and proceeds through a multi-step sequence starting from commercially available acetylacetone. Initially, acetylacetone (**1**) was subjected to chlorination using sulfuryl chloride in toluene at room temperature for 16 h, affording 3-chloroacetylacetone (**2**) as the key electrophilic intermediate. This chlorinated derivative subsequently underwent cyclization with ammonium hydroxide and carbon disulfide in ethanol under reflux conditions, leading to the formation of the thiazole intermediate (**3**) with a moderate yield of 69%.

Parallel to this, 4-sulfamoylbenzoic acid **(4**) was reduced using borane in tetrahydrofuran at 0°C for 19 h to afford the corresponding benzyl alcohol (**5**) in excellent yield (93%). Subsequent oxidation of this alcohol with pyridinium chlorochromate in dichloromethane under reflux for 6 h afforded the aldehyde intermediate (**6**) in 76% yield, serving as a key precursor for the final condensation step. The final chalcone derivative (**7**) was synthesized through a base-catalyzed Claisen–Schmidt condensation between the thiazole intermediate (**3**) and the aldehyde (**6**). The reaction was conducted in ethanol with 60% sodium hydroxide at 0°C for 12 h, facilitating the formation of the α,β-unsaturated carbonyl system characteristic of chalcone scaffolds. The final compound was isolated in good yield (73%), demonstrating the efficiency of the overall synthetic route.

**Reagents and Conditions:** (i) SO_2_Cl_2_, toluene, RT, 16 h; (ii) NH_3_, CS_2_, EtOH, reflux, 6 h; (iii) BH_3_, THF, 0°C, 19 h; (iv) PCC, DCM, reflux, 6 h; (v) 60% NaOH, ethanol, 0°C, 12 h.

The structure of the final compound was characterized by ^1^H NMR, ^13^C NMR, and elemental analysis. The ^1^H NMR spectrum, recorded in DMSO-d_6_, displayed distinct signals consistent with the proposed structure. A downfield singlet appeared at δ 13.64 ppm, corresponding to the thiazole N-H proton, confirming the formation of the thiazole ring. Aromatic protons from the sulfonamide phenyl ring appeared as two doublets at δ 7.93 and δ 7.83 ppm. The olefinic protons of the α,β-unsaturated carbonyl system were observed as two trans-coupled doublets at δ 7.51 and δ 7.41 ppm, indicating the *E*-configuration of the double bond. A singlet at δ 7.23 ppm corresponded to the sulfonamide protons, while the methyl group resonated as a singlet at δ 2.59 ppm. The ^13^C NMR spectrum further supported the structure of compound 7, displaying key carbon resonances in DMSO-d_6_. A characteristic signal appeared at δ 189.50 ppm, corresponding to the thiocarbonyl carbon of the thiazole ring, while the carbonyl carbon of the chalcone moiety resonated at δ 180.17 ppm. Additionally, the methyl group carbon was observed at δ 15.57 ppm, consistent with the presence of the methyl-substituted thiazole.

### 2.2 Biological evaluation

#### 2.2.1 Antiproliferative assay

**FIGURE 4:**
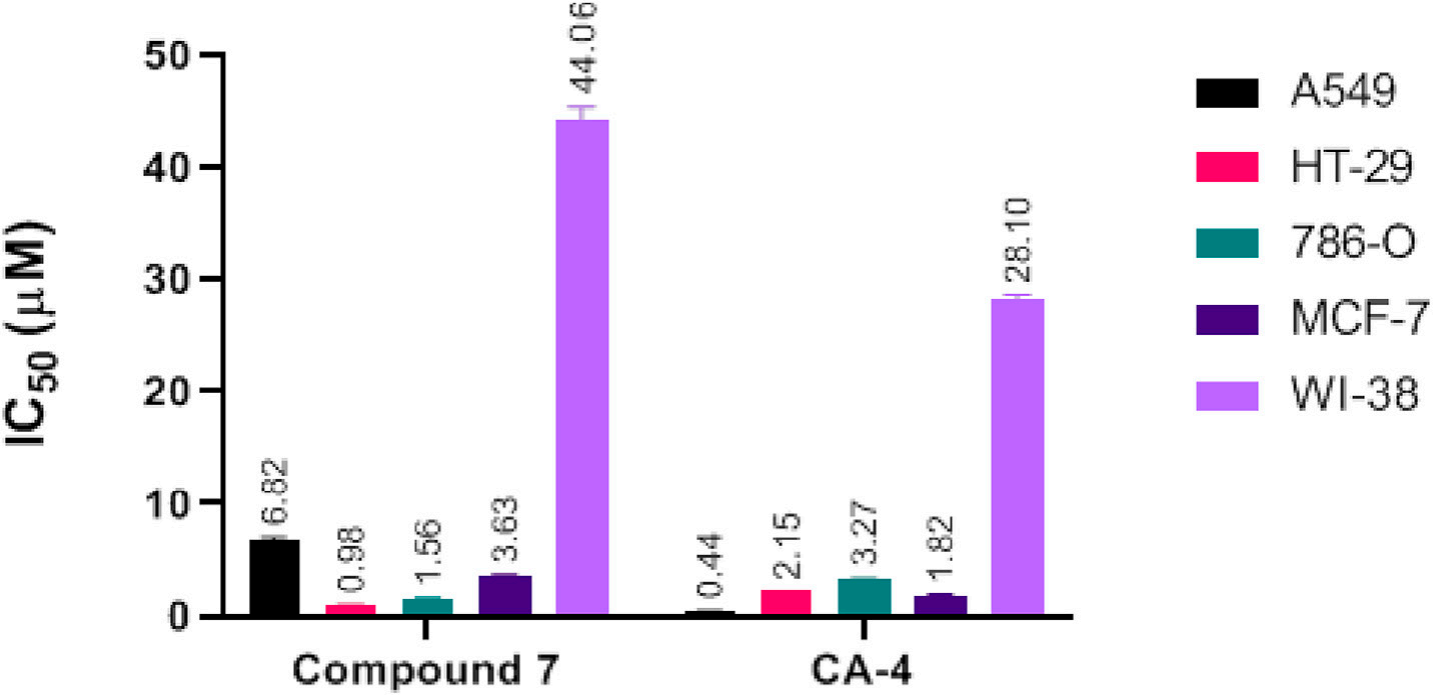
Comparative IC_50_ values of compound **7** and combretastatin A-4 (CA-4) against A549 (lung), HT-29 (colorectal), 786-O (renal), and MCF-7 (breast) cancer cell lines, as well as the WI-38 normal fibroblast cell line, determined by MTT assay. Data are mean ± SEM.

The antiproliferative activity of Compound **7** was evaluated against A549, HT-29, 786-O, and MCF-7 cancer cell lines, as well as the normal WI-38 fibroblast cell line, using the MTT assay and compared with combretastatin A-4 (CA-4) as a reference compound (Huo et al., 2021). As shown in Figure 4, compound **7** exhibited considerable cytotoxic activity across the tested cancer cell lines, with the most pronounced effect observed against the HT-29 colorectal cancer cells, where it achieved an IC_50_ value of 0.98 μM. Notably, this potency surpassed that of CA-4 against the same cell line (IC_50_ = 2.15 μM), indicating a preferential effect of compound **7** toward colorectal carcinoma.

Compound **7** also displayed good activity against 786-O renal cancer cells (IC_50_ = 1.56 μM) and moderate inhibition of A549 lung (IC_50_ = 6.82 μM) and MCF-7 breast cancer cells (IC_50_ = 3.63 μM). In contrast, CA-4 exhibited higher potency against A549 (IC_50_ = 0.44 μM) and MCF-7 cells (IC_50_ = 1.82 μM), confirming its well-known broad cytotoxic profile. However, selectivity analysis revealed that compound **7** had a more favorable therapeutic index. When tested against WI-38 normal fibroblasts, compound **7** showed an IC_50_ of 44.06 μM, resulting in a remarkably high selectivity index (SI) of 44.96 for HT-29 and 28.25 for 786-O cells. These values were significantly higher than those calculated for CA-4, which showed an SI of 13.06 for HT-29 and 8.59 for 786-O.

#### 2.2.2 Effect of compound 7 on tubulin polymerization

To investigate the potential mechanism underlying its antiproliferative activity, compound **7** was evaluated for its ability to inhibit tubulin polymerization (Huo et al., 2021). As presented in Table 1, the compound exhibited notable tubulin polymerization inhibition with an IC_50_ value of 2.72 μM, which is comparable to that of CA-4 (IC_50_ = 2.97 μM). This result suggests that compound **7** may exert its antiproliferative effects, at least in part, through interference with microtubule dynamics, similar to CA-4. It is noteworthy that compound 7 was developed through the structural optimization of our previously reported thiazole–chalcone derivative, Tz, which showed moderate tubulin polymerization inhibition (IC_50_ = 7.78 μM). Compared to **Tz**, compound **7** incorporates a para-substituted sulfonamide group designed to enhance polar interactions within the colchicine-binding site. This modification appears to have significantly improved its tubulin inhibitory potency. The introduction of the electron-withdrawing sulfonamide moiety likely enhances binding affinity by establishing additional hydrogen bonds and reinforcing anchorage within the active site, resulting in a lower IC_50_ value.

**TABLE 1:** Tubulin polymerization inhibition (IC_50_, μM) of compound 7 and combretastatin A-4, as determined by an *in vitro* assay. Data are presented as mean ± SEM.

Compound	Tubulin polymerization inhibition IC_50_ ± SEM (μM)
**7**	2.72 ± 0.16
**CA-4**	2.97 ± 0.09

#### 2.2.3 Evaluation of carbonic anhydrase I, II, IX, and XII inhibition

To further investigate the biological profile of compound **7**, its inhibitory activity against a panel of human carbonic anhydrase (CA) isoforms was evaluated, including the physiologically relevant isoforms CA I and CA II, as well as the tumor-associated isoforms CA IX and CA XII (Albelwi et al., 2024). As summarized in Table 2, compound **7** exhibited selective and potent inhibition toward the tumor-associated CA IX isoform with an IC_50_ value of 0.021 ± 0.004 μM, outperforming the reference inhibitors acetazolamide (AAZ) and SLC-0111, which showed IC_50_ values of 0.105 ± 0.01 μM and 0.048 ± 0.006 μM, respectively. This remarkable potency against CA IX suggests that compound **7** may effectively target the hypoxic microenvironment of solid tumors, where CA IX is overexpressed and contributes to tumor progression, invasion, and metastasis.

**TABLE 2:** IC_50_ values (µM) of compound **7**, acetazolamide (AAZ), and SLC-0111 against CA isoforms I, II, IX, and XII. Data are expressed as mean ± SEM.

Compound	CA inhibition IC_50_ (μM) ±SEM			
	CA I	CA II	CA IX	CA XII
**7**	1.90 ± 0.25	0.381 ± 0.045	0.021 ± 0.004	0.114 ± 0.012
**AAZ**	0.367 ± 0.02	0.153 ± 0.01	0.105 ± 0.01	0.029 ± 0.001
**SLC-0111**	1.36 ± 0.07	0.498 ± 0.04	0.048 ± 0.006	0.096 ± 0.008

Additionally, compound **7** showed potent inhibition of CA XII (IC_50_ = 0.114 ± 0.012 μM), another tumor-associated isoform, indicating a potential dual inhibitory effect that could enhance anticancer efficacy. In contrast, its activity against the ubiquitous and off-target isoforms CA I and CA II was significantly lower, with IC_50_ values of 1.90 ± 0.25 μM and 0.381 ± 0.045 μM, respectively. This selectivity profile is favorable compared to AAZ, which displayed strong inhibition of CA I (IC_50_ = 0.367 ± 0.02 μM) and CA II (IC_50_ = 0.153 ± 0.01 μM), raising concerns about potential off-target effects and toxicity.

To better illustrate this selectivity, selectivity indices (SI) were calculated as the ratio of IC_50_ values for the off-target isoforms to those for the tumor-associated isoforms, as presented in Table 3. Compound **7** demonstrated outstanding selectivity toward CA IX, with SI values of CA I/CA IX = 90.5 and CA II/CA IX = 18.1. These values markedly exceeded those of AAZ (CA I/CA IX = 3.5, CA II/CA IX = 1.5) and SLC-0111 (CA I/CA IX = 28.3, CA II/CA IX = 10.4), confirming the superior tumor-targeting potential of compound **7**. Moreover, the CA I/CA XII selectivity ratio of compound **7** reached 16.7, surpassing AAZ (12.7) and closely aligning with SLC-0111 (14.2), further supporting its preferential targeting of tumor-associated isoforms.

**TABLE 3:** Selectivity indices (SI) of compound **7**, acetazolamide (AAZ), and SLC-0111 for tumor-associated CA IX and CA XII over off-target isoforms CA I and CA II.

Compound	Selectivity index (SI)			
	CA I/CA IX	CA II/CA IX	CA I/CA XII	CA II/CA XII
**7**	90.5	18.1	16.7	3.3
**AAZ**	3.5	1.5	12.7	5.3
**SLC-0111**	28.3	10.4	14.2	5.2

Interestingly, this strong and selective inhibition of CA IX correlates well with the MTT assay results, where compound **7** demonstrated its most potent antiproliferative activity against HT-29 (IC_50_ = 0.98 μM) and 786-O (IC_50_ = 1.56 μM) cancer cell lines. Both of these tumor types are known to constitutively express high levels of CA IX, suggesting that the remarkable cytotoxicity of Compound 7 toward these cells may be partially attributed to its potent inhibition of CA IX. This mechanistic link further supports the potential of Compound 7 to act selectively against CA IX-expressing tumors, offering a targeted approach to anticancer therapy.

#### 2.2.4 Effects of compound **7** on p53, Bax and Bcl-2 protein expression levels

To further explore the potential apoptotic mechanism underlying the antiproliferative effects of compound **7**, its impact on key apoptosis-related proteins—p53, Bax, and Bcl-2—was investigated in HT-29 cells (Ahmed et al., 2024). As shown in Table 4, treatment with compound **7** resulted in a substantial upregulation of the tumor suppressor protein p53, increasing its concentration from 150.66 ± 3.33 pg/mL in the DMSO-treated control to 1,224.00 ± 6.67 pg/mL, representing an 8.12-fold elevation. This significant induction of p53 suggests activation of a p53-mediated apoptotic pathway, which is known to play a critical role in controlling cell cycle arrest and promoting apoptosis in response to cellular stress and DNA damage. Similarly, compound **7** significantly increased the expression of the pro-apoptotic protein Bax, resulting in a 5.73-fold increase in its level compared to the control. Bax concentration reached 392.25 ± 14.58 pg/mL, up from 68.50 ± 1.67 pg/mL in DMSO-treated cells. The elevation of Bax further supports the pro-apoptotic effect of compound **7**, as Bax promotes mitochondrial outer membrane permeabilization, leading to the release of cytochrome c and caspase activation. Conversely, the anti-apoptotic protein Bcl-2 was significantly downregulated following treatment with compound 7. Bcl-2 concentration decreased from 19.25 ± 0.43 pg/mL in control cells to 6.212 ± 0.093 pg/mL, reflecting a 0.323-fold change. This notable reduction in Bcl-2 levels favors the apoptotic process by diminishing its protective effect against mitochondrial-mediated apoptosis.

**TABLE 4:** Effects of Compound **7** on p53, Bax, and Bcl-2 protein expression levels in HT-29 cells.

Compound	P53		Bax		Bcl-2	
	Conc (pg/mL)	Fold change	Conc (pg/mL)	Fold change	Conc (pg/mL)	Fold change
**7/HT-29**	1,224.00 ± 6.67	8.12	392.25 ± 14.58	5.73	6.212 ± 0.093	0.323
**DMSO/HT-29**	150.66 ± 3.33	1	68.50 ± 1.67	1	19.25 ± 0.43	1

Collectively, these findings demonstrate that compound **7** effectively shifts the Bax/Bcl-2 ratio in favor of apoptosis and activates p53-mediated pathways in HT-29 cells. The combined upregulation of p53 and Bax, along with the suppression of Bcl-2, provides strong evidence that compound **7** induces apoptosis as a major mechanism contributing to its potent antiproliferative activity against colorectal cancer cells.

#### 2.2.5 Effects of compound 7 on caspase-3 and caspase-9 activities

To further confirm the apoptotic pathway induced by compound **7** in HT-29 cells, the activation of caspase-3 and caspase-9—key executioner and initiator caspases, respectively—was evaluated (Abdel-Motaal et al., 2024). As detailed in Table 5, treatment with compound **7** resulted in a significant increase in caspase-3 levels, reaching 511.27 ± 13.18 pg/mL, compared to 57.64 ± 1.36 pg/mL in DMSO-treated control cells. This represents an 8.87-fold increase, indicating robust activation of the downstream effector caspase, which is essential for the execution phase of apoptosis. In parallel, caspase-9, a central mediator of the intrinsic mitochondrial apoptotic pathway, was significantly activated following treatment with compound 7. The caspase-9 level increased from 5.09 ± 0.26 ng/mL in control cells to 49.39 ± 1.13 ng/mL, corresponding to a 9.70-fold elevation. This pronounced increase strongly suggests that compound **7** triggers the intrinsic apoptotic pathway, likely *via* mitochondrial damage and cytochrome c release, leading to caspase-9 activation and subsequent caspase-3 cleavage.

**TABLE 5:** Effects of **Compound 7** on caspase-3 and caspase-9 activities in HT-29 cells.

Compound	Caspase 3		Caspase-9	
	Conc (pg/mL)	Fold change	Conc (ng/mL)	Fold change
**7/HT-29**	511.27 ± 13.18	8.87	49.39 ± 1.13	9.70
**DMSO/HT-29**	57.64 ± 1.36	1	5.09 ± 0.26	1

Together, the significant activation of both caspase-9 and caspase-3 provides compelling evidence that compound **7** induces apoptosis in HT-29 cells through the intrinsic mitochondrial pathway. These findings are consistent with the observed upregulation of p53 and Bax and the downregulation of Bcl-2, reinforcing the role of mitochondrial-mediated apoptosis as a key mechanism underlying the potent anticancer activity of compound **7**.

### 2.3 Molecular modeling studies

#### 2.3.1 Molecular docking studies

##### 2.3.1.1 Docking of compound **7** into the colchicine binding site of tubulin

**FIGURE 5:**
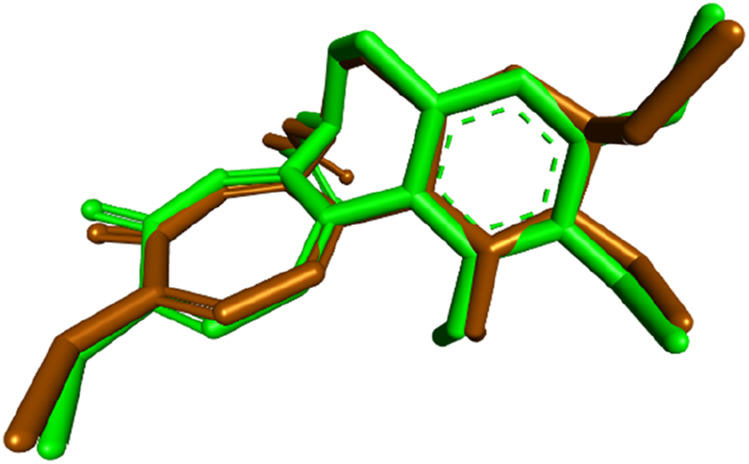
Superimposition of the redocked (brown) and cocrystallized (green) poses of colchicine within the binding site of tubulin.

To gain further insights into the molecular basis of the tubulin polymerization inhibition observed for compound **7**, molecular docking studies were conducted targeting the colchicine-binding site of tubulin. The crystal structure of the tubulin–colchicine complex (PDB ID: 4O2B) was utilized for this purpose (Boichuk et al., 2021). Docking was performed using AutoDock Vina (Trott and Olson, 2010), and the resulting ligand–protein interactions were visualized using Discovery Studio Visualizer. Before docking the test compound, the docking protocol was validated by redocking the co-crystallized ligand colchicine into its native binding site. The redocked pose showed a binding affinity of −8.5 kcal/mol and a root-mean-square deviation (RMSD) of 0.7173 Å compared to the crystallographic orientation, confirming the reliability of the docking approach. The superimposition of the docked and experimental poses is illustrated in Figure 5.

**FIGURE 6:**
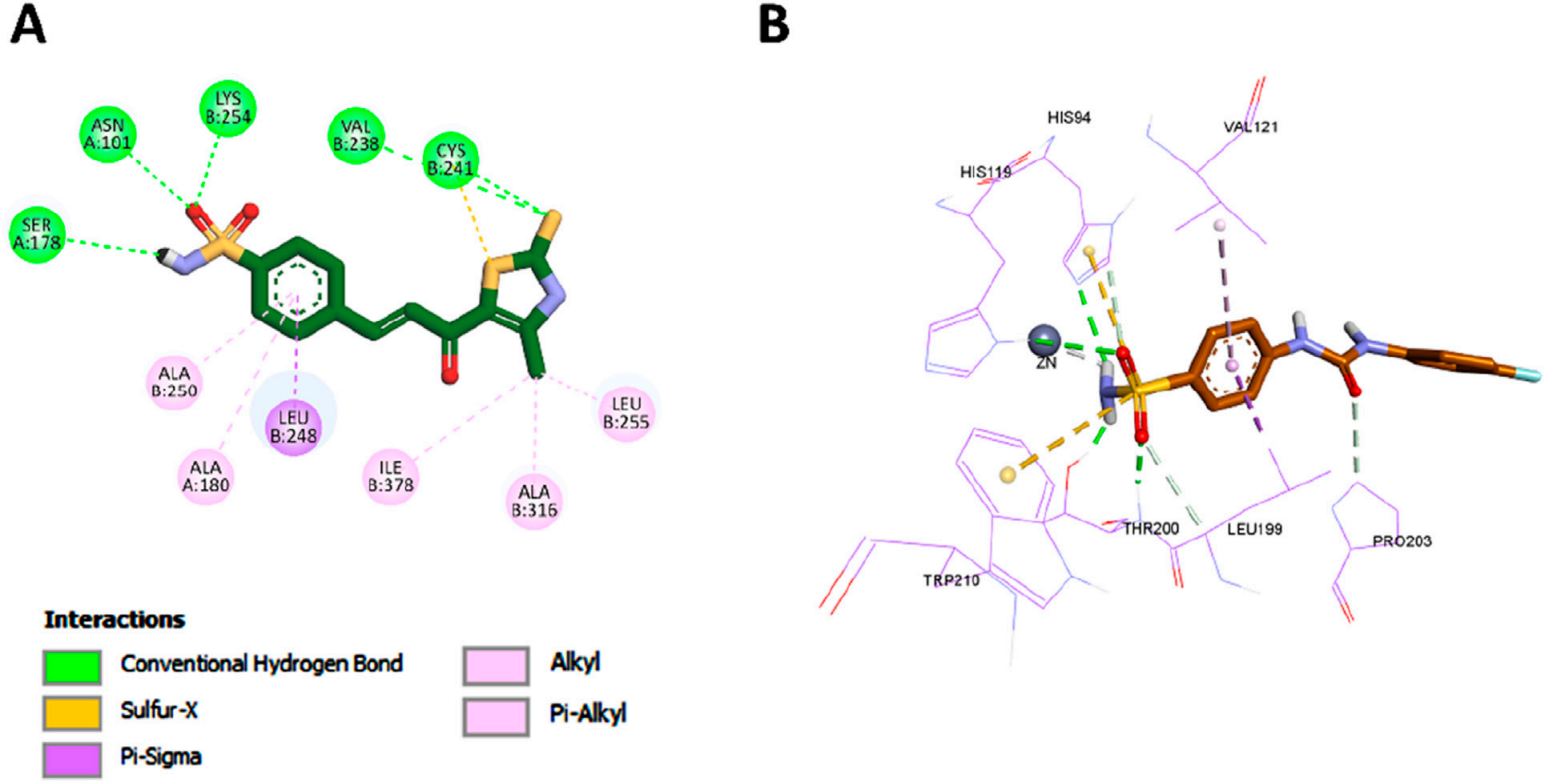
2D **(A)** and 3D **(B)** interactions of compound **7** within the colchicine binding site of tubulin.

**FIGURE 7:**
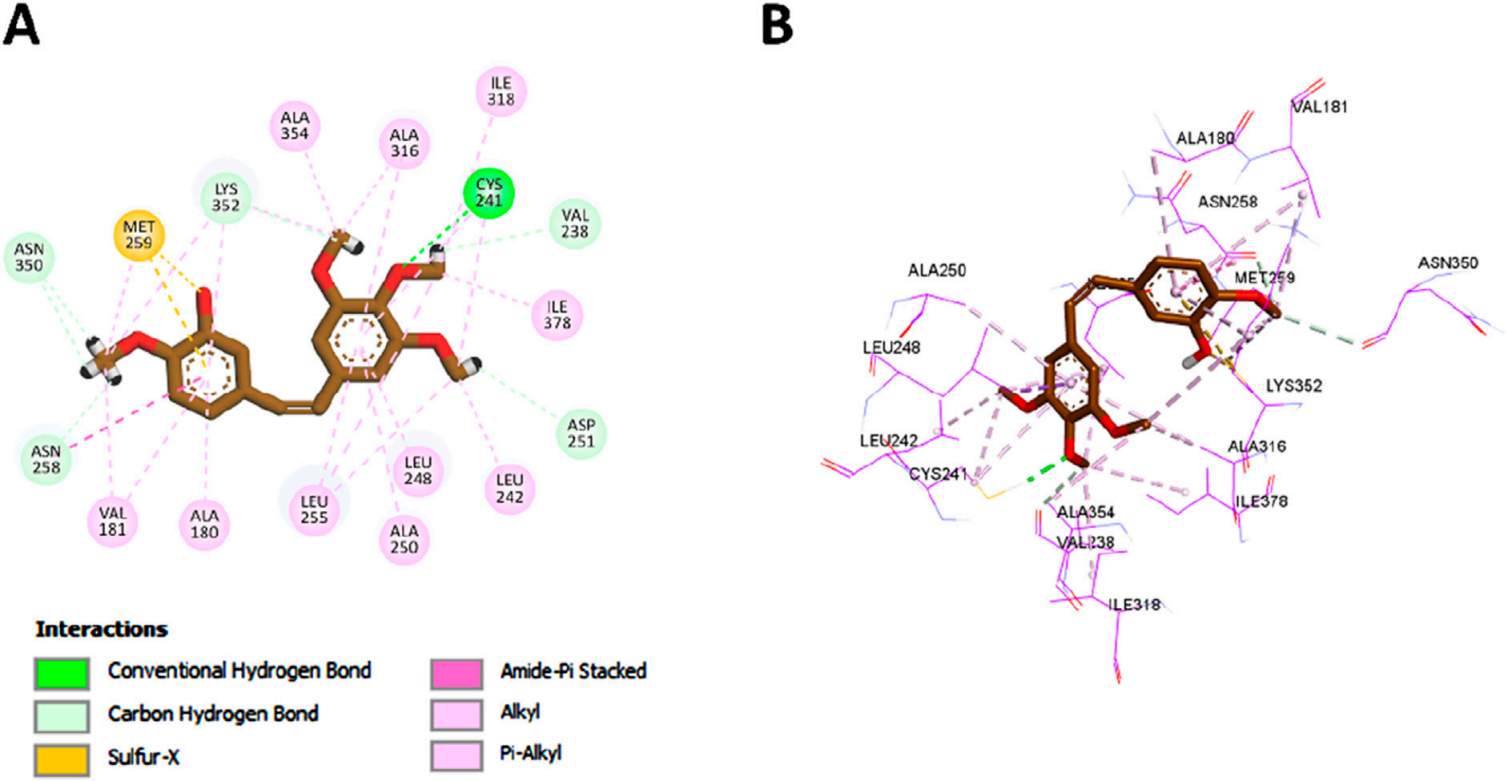
2D **(A)** and 3D **(B)** interactions of compound CA-4 within colchicine binding site of tubulin.

**FIGURE 8:**
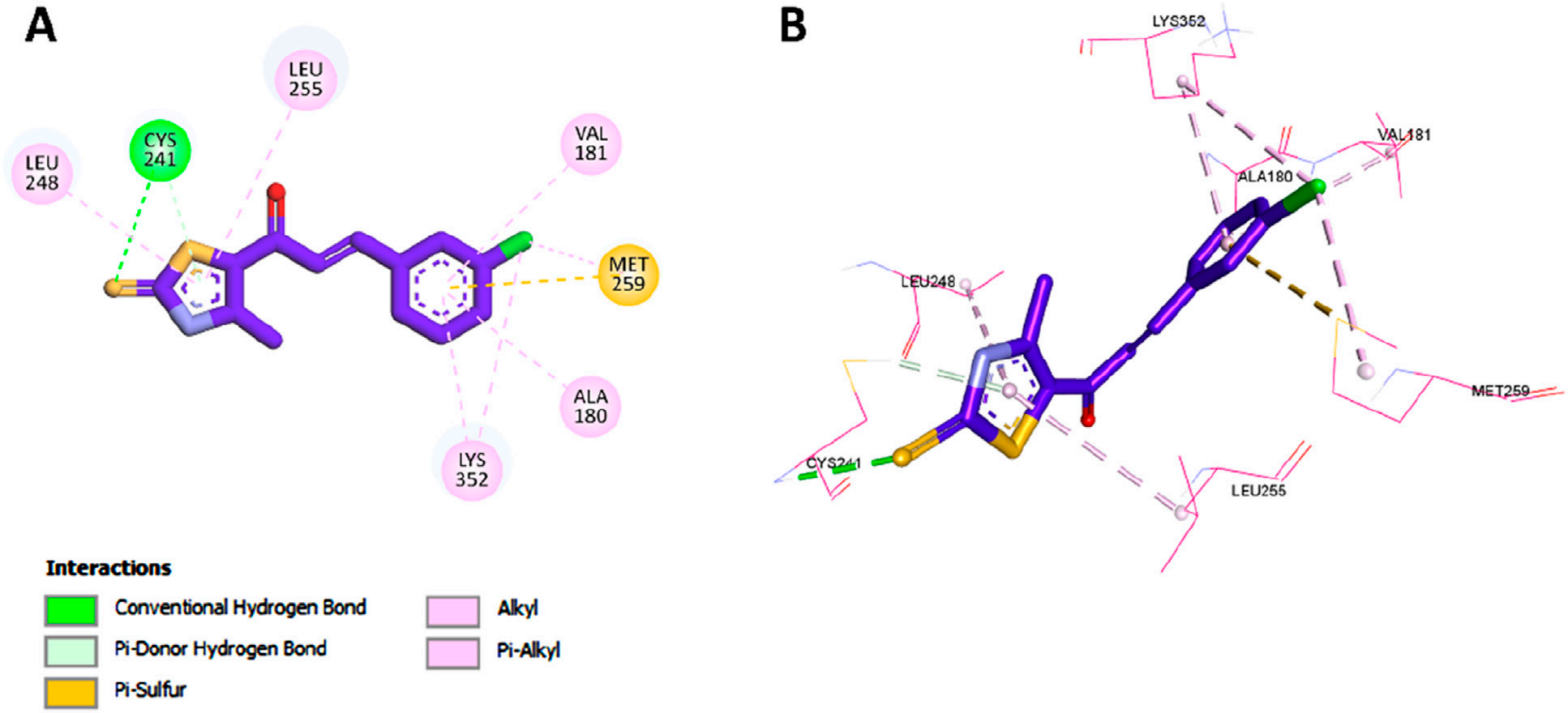
2D **(A)** and 3D **(B)** interactions of compound **Tz** within the colchicine binding site of tubulin.

Compound **7** was subsequently docked into the colchicine-binding site and yielded a binding affinity of −9.8 kcal/mol, which is slightly more favorable than that of the reference compound combretastatin A-4 (CA-4, –9.6 kcal/mol), suggesting a strong interaction with the target site. As depicted in Figure 6, compound **7** exhibited several critical interactions that underlie its high binding affinity. Notably, the thiocarbonyl group formed a classical hydrogen bond with Cys241, an interaction previously highlighted as essential for the tight binding of tubulin inhibitors such as colchicine and CA-4. In addition, compound **7** retained several important hydrophobic interactions observed in both CA-4 and **Tz**, including contacts with Leu255, Leu248, Ala180, and Val181 (Figures 7, 8).

Beyond these conserved interactions, compound **7** introduced additional hydrogen bonding contacts not seen in CA-4 or **Tz**. Specifically, the thiocarbonyl group formed hydrogen bonds with Val238, while the sulfonamide group at the para position of the phenyl ring formed new hydrogen bonds with Ser178, Asn101, and Lys254. These unique interactions not only enhance the binding affinity but also serve to anchor compound **7** more securely within the colchicine-binding pocket, thereby stabilizing the ligand–protein complex. The presence of the sulfonamide moiety, in particular, appears to reinforce molecular recognition and may contribute to the slightly superior tubulin inhibition profile of compound **7** compared to both CA-4 and the previously reported **Tz**.

##### 2.3.1.2 Docking of compound **7** into the carbonic anhydrase IX active site

**FIGURE 9:**
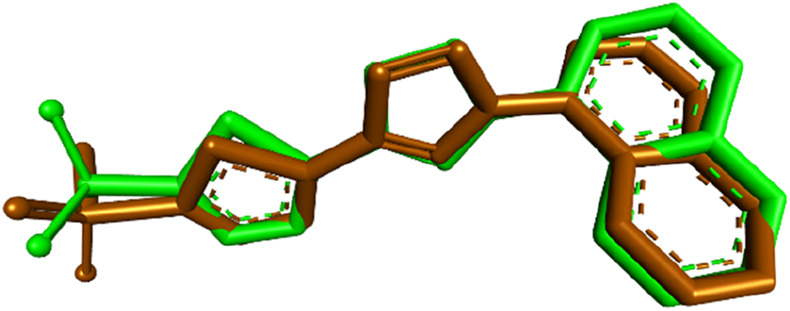
Superimposition of the redocked (brown) and cocrystallized (green) poses of the native ligand within the CAIX active site.

To complement the experimental findings on the potent and selective inhibition of CA IX by compound **7**, molecular docking was performed to explore its binding interactions within the active site of CA IX (PDB ID: 5FL4). The docking protocol was first validated by redocking the co-crystallized ligand into the CA IX crystal structure, resulting in a binding affinity of −6.8 kcal/mol and an RMSD of 0.8532 Å relative to the crystallographic pose, as illustrated in Figure 9. This result confirmed the accuracy of the docking methodology.

Upon docking, compound **7** exhibited a binding affinity of −8.1 kcal/mol, consistent with its experimentally observed submicromolar inhibition of CA IX. The sulfonamide moiety of compound **7** played a central role in anchoring the molecule within the active site. Its amino group directly coordinated with the catalytic zinc ion and formed classical hydrogen bonds with key residues His119 and Thr200. Additionally, the sulfonyl group contributed two more hydrogen bonds with these same residues, further stabilizing the complex. The sulfur atom of the sulfonamide also participated in π–sulfur interactions with His94 and Trp210, which further contributed to the overall affinity of the ligand within the active site.

**FIGURE 10:**
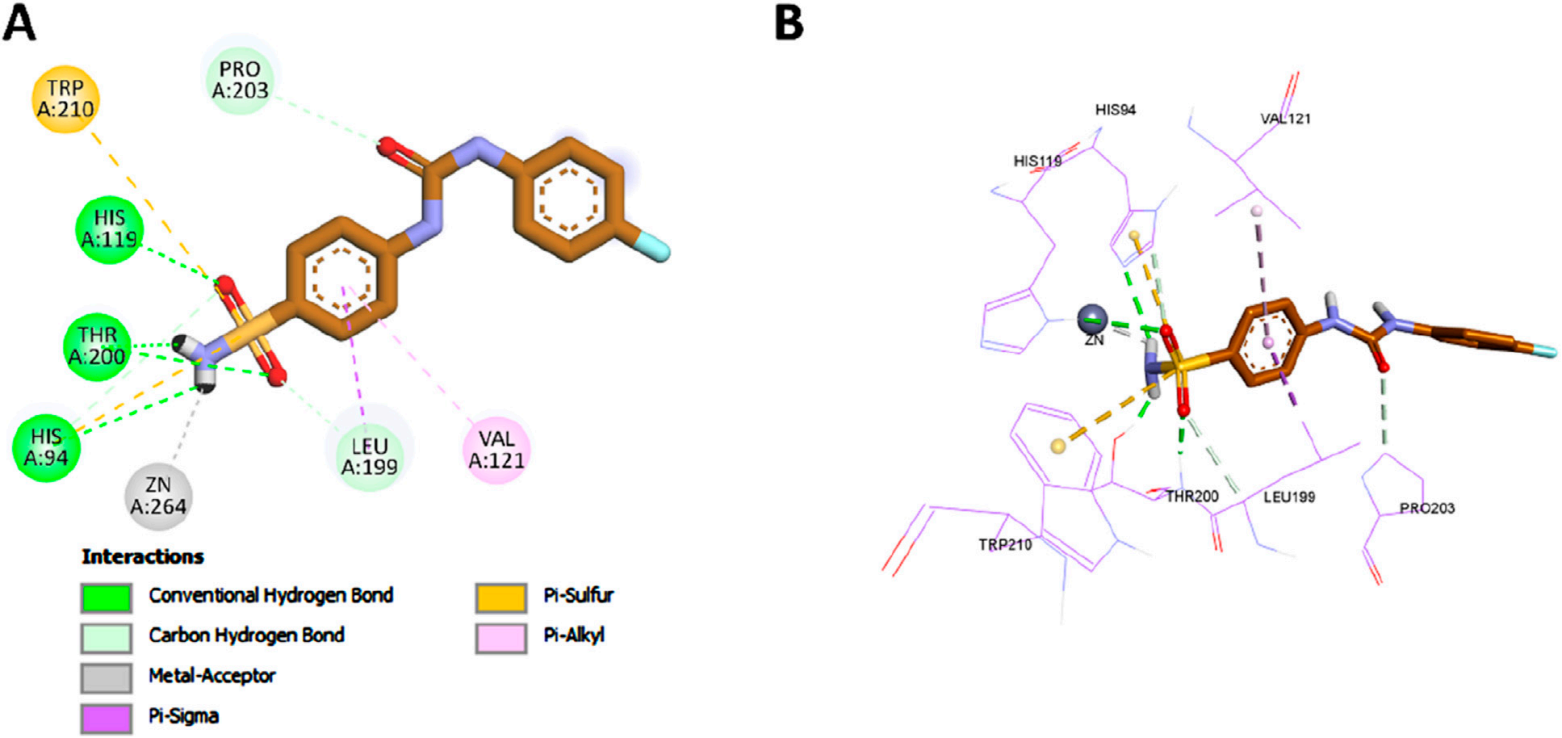
2D **(A)** and 3D **(B)** interactions of SLC-0111 within the CA IX active site.

The docking pose further revealed that the phenyl ring of compound **7** contributed to hydrophobic stabilization through a π–σ interaction with Leu199 and a π–alkyl interaction with Val121. These interactions are comparable to those observed with the reference inhibitor SLC-0111, which also employs a sulfonamide pharmacophore and a hydrophobic tail (Figure 10). Additionally, the carbonyl of the chalcone linker of compound **7** formed a non-classical carbon–hydrogen bond with Pro203, mirroring an analogous interaction observed with the urea carbonyl of SLC-0111.

**FIGURE 11:**
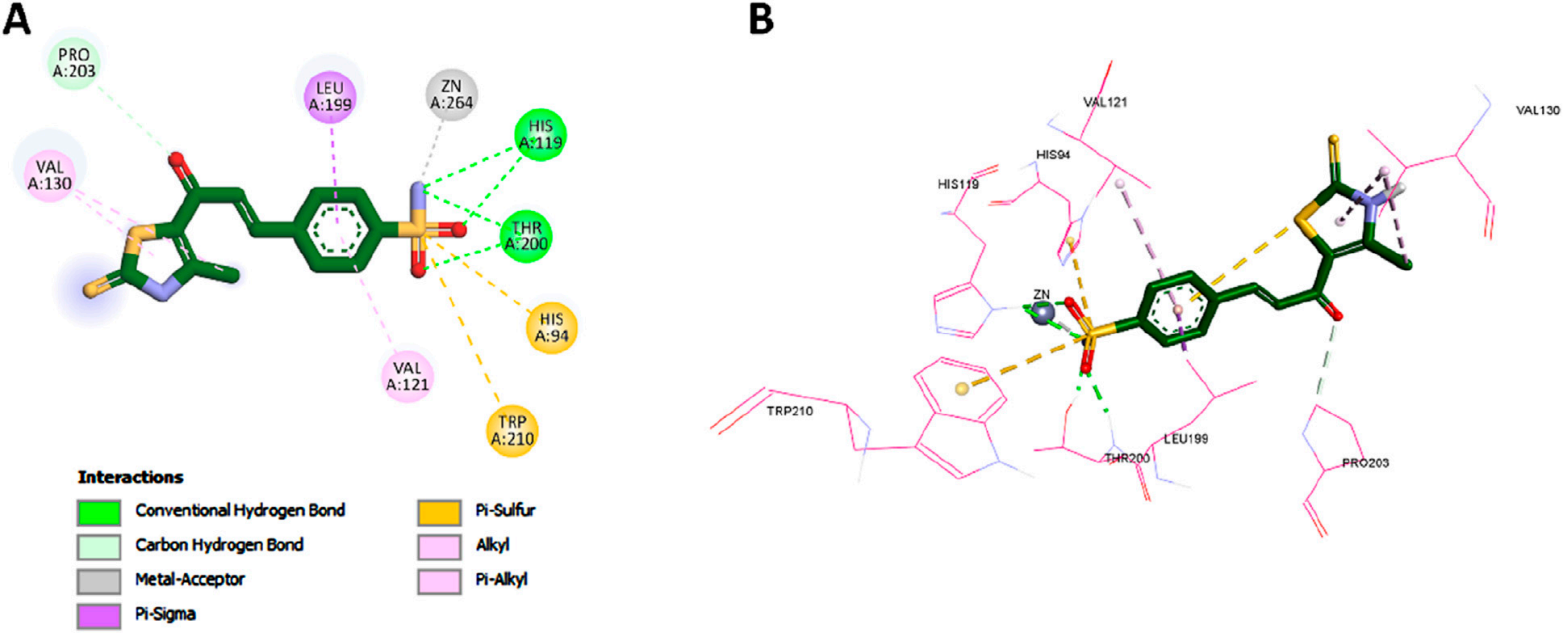
2D **(A)** and 3D **(B)** interactions of compound **7** within the CA IX active site.

A notable structural divergence between the two compounds lies in the tail region: compound **7** features a thiazole ring bearing a methyl substituent, whereas SLC-0111 incorporates a fluorophenyl moiety. This thiazole ring engaged in a π–alkyl interaction with Val130, and its methyl group formed an additional alkyl interaction with the same residue. These unique contacts are visualized in Figure 11 and likely contribute to anchoring compound **7** more firmly within the CA IX active site, thereby enhancing its binding strength and selectivity. Overall, the docking results provide a structural rationale for the experimentally observed potency and specificity of compound **7**, reinforcing its promise as a dual-targeted anticancer agent.

#### 2.3.2 Drug likeness and ADMET predictions

The drug-likeness and ADMET profile of compound **7** were evaluated using SwissADME (Daina et al., 2017) to assess its suitability as an orally bioavailable anticancer agent. With a molecular weight of 340.44 g/mol, four rotatable bonds, and acceptable aromatic content, compound **7** satisfies key drug-likeness criteria, including Lipinski, Ghose, and Muegge rules. Its topological polar surface area (TPSA) of 161.73 Å^2^, though exceeding classical thresholds, is consistent with enzyme-targeted scaffolds, where elevated polarity enhances binding within hydrophilic active sites, such as the zinc-containing pocket of CA IX.

The compound exhibits a favorable lipophilicity profile, with a consensus Log P_o_w of 2.20, indicating a well-balanced hydrophilic–lipophilic character conducive to membrane permeability and solubility. It is predicted to be soluble or moderately soluble across three models, supporting its potential for oral administration.

Although compound **7** shows low predicted gastrointestinal absorption, this may reduce systemic off-target exposure while favoring localized tumor accumulation—particularly when paired with targeted delivery platforms. The compound does not cross the blood–brain barrier and is not a P-gp substrate, limiting CNS exposure and efflux-related resistance. It is predicted to inhibit several CYP450 isoforms, a property that warrants attention during development but may be beneficial for prolonging systemic half-life under controlled regimens.

**FIGURE 12:**
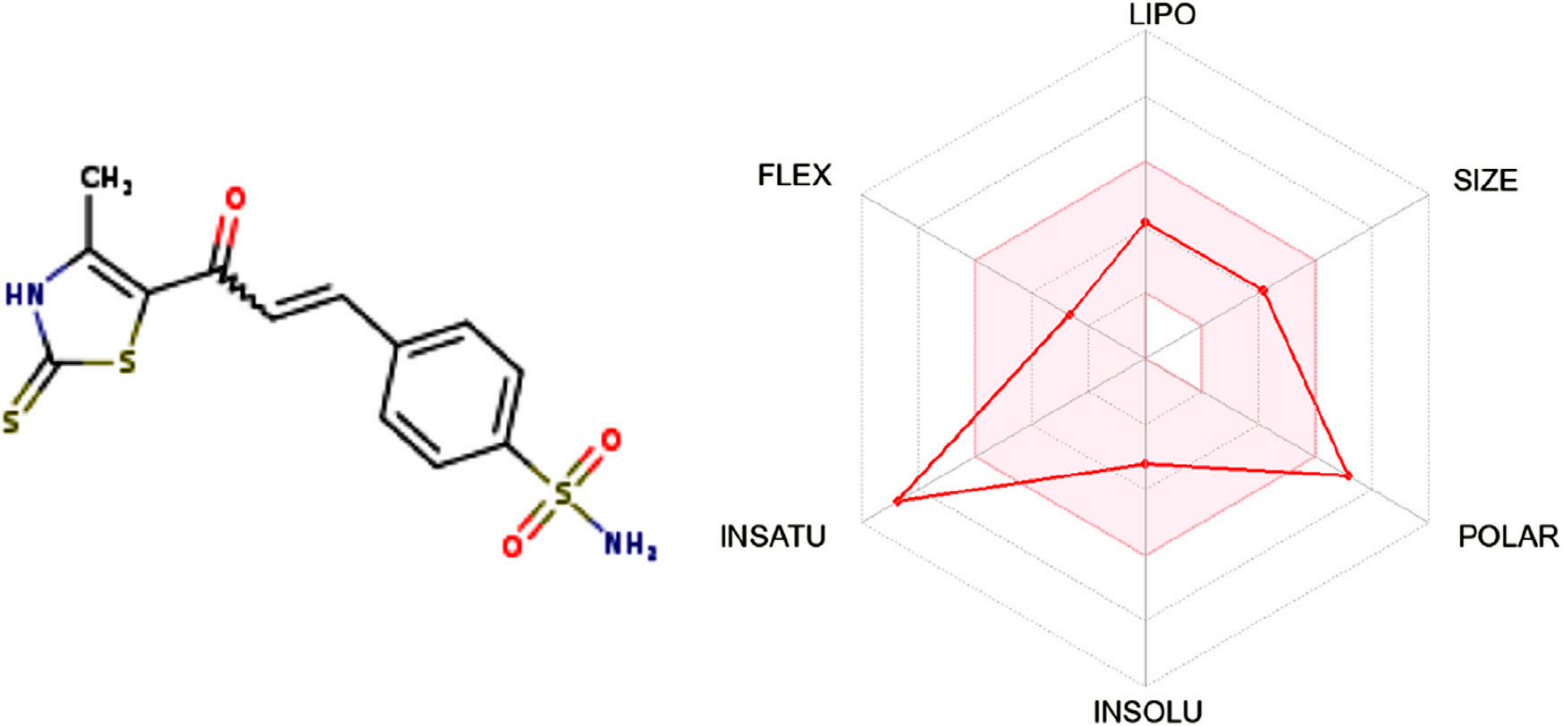
Bioavailability radar of compound **7** showing overall drug-like properties with minor deviations in polarity and saturation.

The bioavailability score (0.55) and synthetic accessibility (3.30) suggest good oral drug potential and moderate ease of synthesis. Medicinal chemistry filters report no PAINS alerts and only two Brenk flags, both associated with functional groups essential for biological activity. The bioavailability radar, as depicted in Figure 12, highlights optimal values for size, flexibility, solubility, and lipophilicity, with slight deviations in polarity and unsaturation, reflecting the structural features necessary for target engagement.

In summary, compound **7** exhibits a favorable drug-likeness and ADMET profile. Its physicochemical and pharmacokinetic attributes, combined with its dual-targeting activity, support its potential as a promising lead in anticancer drug development.

#### 2.3.3 DFT calculations

##### 2.3.3.1 Geometrical structure and frontier molecular orbitals (FMOs)

**FIGURE 13:**
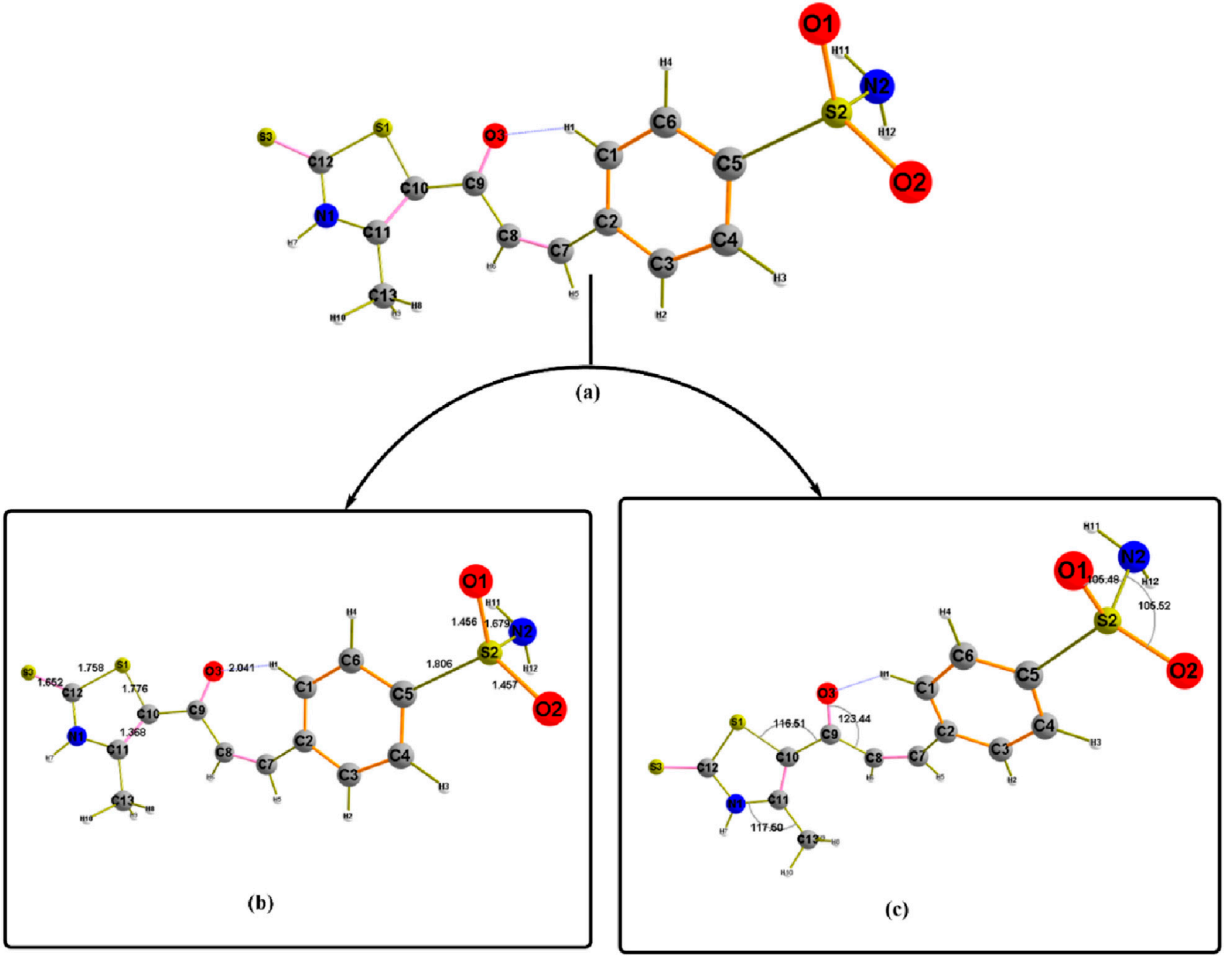
Geometrical structure of compound **7 (a)** labeled with **(b)** bond lengths, **(c)** bond angles.

Figure 13 presents the investigated geometrical structural convergence of compound **7** in its ground state, utilizing the same theoretical level. To develop a deeper understanding of the conformational behavior of this structure, it is essential to examine two fundamental structural properties: bond lengths and bond angles. These parameters provide significant insights into the molecular geometry and its deviation from planarity. The compound’s deviation from a completely planar structure can be attributed to the orientation of its sulfonamide group (SO_2_NH_2_), which is positioned in C5 of the parent part. This spatial arrangement influences the overall stability and electronic distribution within the molecule. Additionally, a CO group at the molecular core plays a pivotal role in inducing a bent geometry. This CO group not only acts as the central point of molecular bending but also forms two hydrogen bonds with the H1 atom, with estimated bond distances of 2.041 Å. The formation of these H-bonds is a critical factor in stabilizing the optimized molecular structure. An important aspect of this structural stability is the validation of the strength of hydrogen bond formation. Other bond length values were estimated for C5-S2 and C12-S3 at 1.806 Å and 1.662 Å, respectively. This difference is attributed to the bond order between atoms. A very slight difference was observed in S2-O1 and S2-O2, where the geometrical environment is similar, facilitating a symmetric electronic distribution with the NH_2_ group. Furthermore, analyzing the bond angle values provides additional clarity regarding the planarity behavior of the atoms within the structure. The bond angles help assess the degree of similarity in the molecular environment surrounding specific atoms. For instance, the angles C11-C13-N1 and C9-N10-S1 measured 117.50° and 116.21°, respectively. This finding suggests a relatively consistent spatial arrangement that contributes to the overall molecular geometry. The angles around the SO2 group also export the same stability results. These findings collectively enhance our understanding of the compound’s structural characteristics and contribute to predicting its stability and reactivity in various chemical contexts.

**FIGURE 14:**
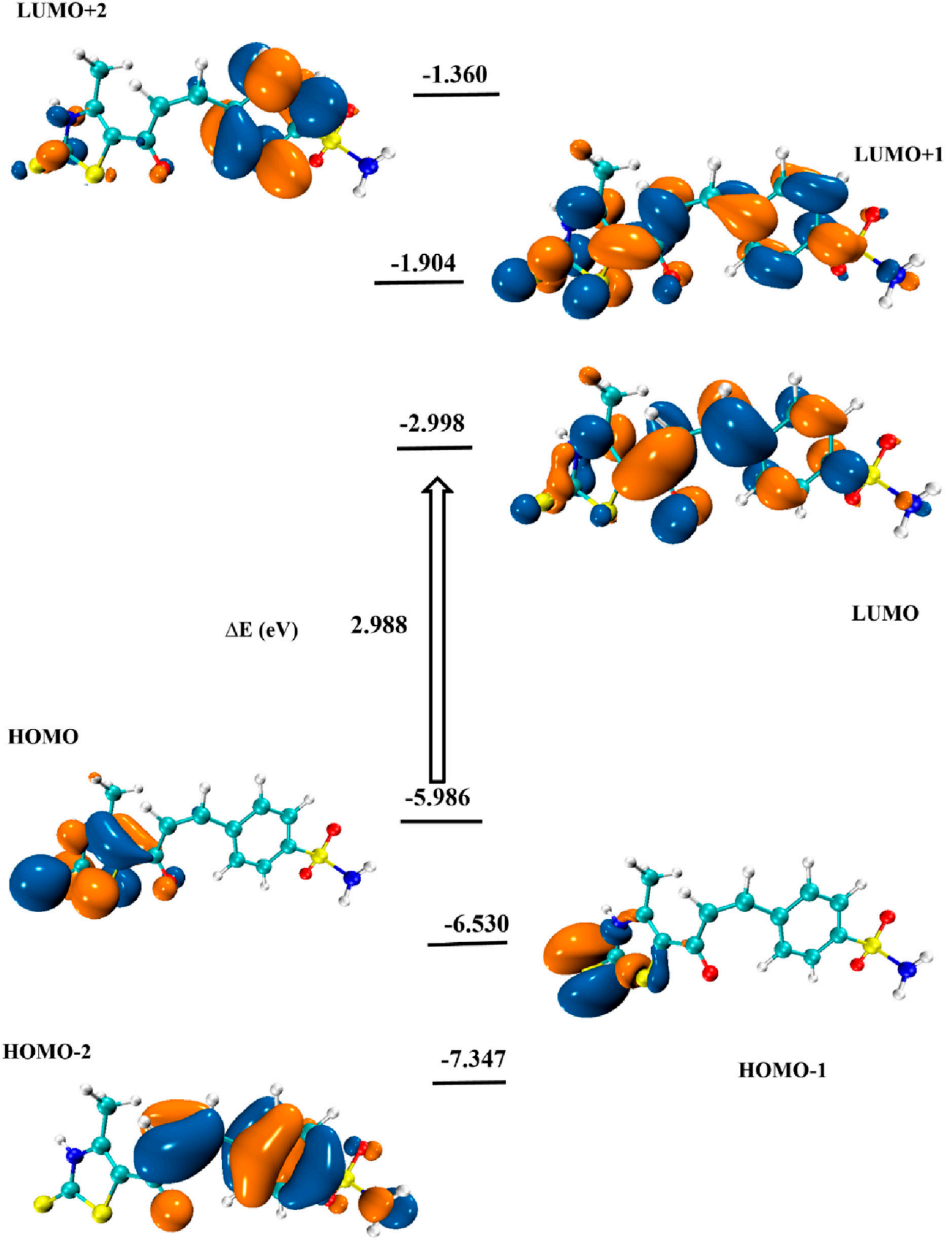
Energy excitation levels with energy values (eV) for compound **7**.

To accurately predict the stability and reactivity of various molecular systems, a comprehensive analysis of the frontier molecular orbitals (FMOs) is essential. These orbitals play a crucial role in determining the electronic properties of a compound, influencing its chemical behavior, interaction potential, and overall stability in different environments. Figure 14 illustrates the energy distribution of the most significant molecular orbitals for the optimized gaseous-phase structure, including HOMO-2, HOMO-1, HOMO, LUMO, LUMO+1, and LUMO+2. By examining these orbitals, valuable insights can be gained into the electronic transitions and potential reactivity sites within the molecule. One of the key factors in assessing the molecular stability of compound **7** is the energy gap (ΔE) between the highest occupied molecular orbital (HOMO) and the lowest unoccupied molecular orbital (LUMO). In this case, the calculated energy gap of 2.988 eV serves as a strong indicator of molecular stability, as a larger gap generally correlates with lower reactivity and greater resistance to electronic excitation. Furthermore, the distribution and contribution of molecular orbitals across different regions of the molecule provide additional evidence supporting the stability of both ground and excited states. The localization of FMOs predominantly around the thiazole ring and carbonyl functional groups suggests a significant role of these moieties in the electronic transitions of the molecule. This observation aligns with the concept of successive donor-acceptor interactions occurring in the excited states, where electron density shifts between these key functional groups. Such interactions are fundamental in determining the electronic excitation pathways and potential photochemical behavior of the compound.

##### 2.3.3.2 UV–vis electronic spectra by TD-DFT method

**FIGURE 15:**
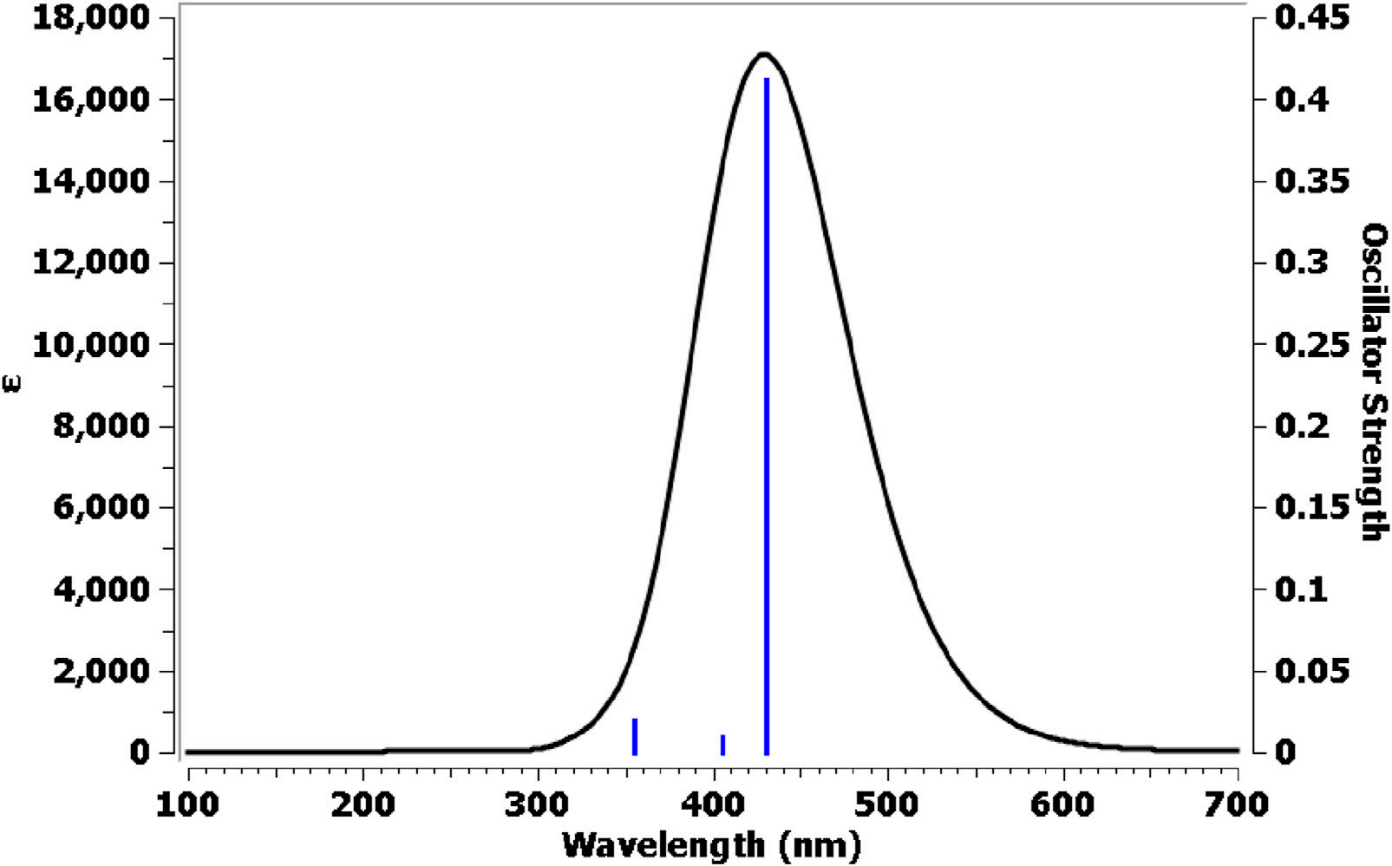
UV–Vis electronic absorption spectra for compound **7**.

To comprehensively analyze the electronic properties of compound **7**, the time-dependent density functional theory (TD-DFT) method, combined with the conductor-like polarizable continuum model (CPCM), was employed to account for solvation effects. These computational approaches enable an accurate description of the molecule’s electronic behavior in solution, providing valuable insights into its optical absorption characteristics. The calculations were performed using the Gaussian 09 software, maintaining the default parameter settings for TD-DFT simulations. To capture the essential electronic transitions, the number of excited states was set to Nstate = 6, ensuring a detailed evaluation of the lowest six electronic states. Figure 15 presents three distinct transition bands corresponding to the observed electronic excitations. The Gaussian calculation’s LOG file confirms the presence of a strong singlet absorption band in the first electronic transition, attributed to an n-π* transition. This transition occurs with a significant contribution of 70%, an excitation energy of 2.88 eV, and a corresponding maximum absorption wavelength (λ_max_) of 430 nm. The HOMO→LUMO excitation predominantly drives this electronic transition, highlighting the participation of non-bonding (n) orbitals in the excitation process. A critical parameter in determining the intensity and probability of electronic transitions is the oscillator strength (F), which provides insight into the transition dipole moment and the likelihood of photon absorption. For the first electronic transition, the oscillator strength is calculated as 0.411, indicating a strong electronic transition efficiency from the ground state to the excited state (LUMO). This higher F value suggests a significant electronic coupling, facilitating effective absorption in the UV-Vis spectrum. Furthermore, Table 6 outlines additional electronic transitions, particularly those involving excitations from HOMO-1 to LUMO and HOMO-2 to LUMO. These transitions exhibit comparable percentage contributions to the overall absorption spectrum, signifying their relevance in the electronic excitation process. However, their respective oscillator strength (F) values differ, reflecting variations in electron transition probabilities between different molecular orbitals. The variations in F values further emphasize the differential ability of electronic transitions based on the spatial and energetic alignment of frontier orbitals.

**TABLE 6:** Excitation energies, maximum wavelengths, oscillator strengths, and % orbital contribution for compound **7**.

Spectral line number	Excitation energy (eV)	λ_max_ (nm)	F	Type of transition	% Orbital contribution
1	2.88	430	0.411	HOMO→LUMO	70
2	3.06	405	0.01	HOMO-1→LUMO	66
3	3.49	355	0.019	HOMO-2→LUMO	55

##### 2.3.3.3 Electron localization function (ELF)

**FIGURE 16:**
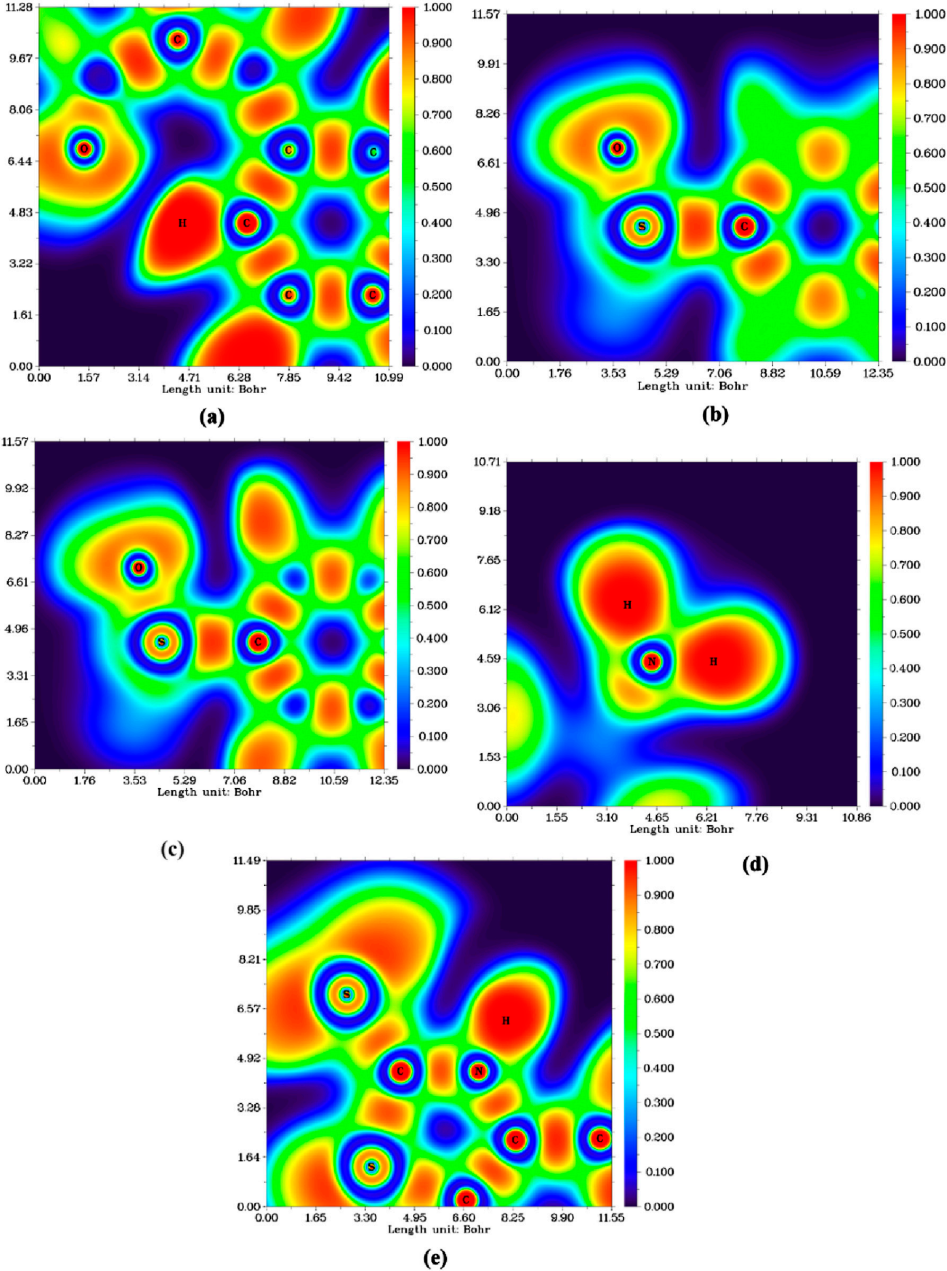
Electron localization function (ELF) colored map of compound **7 (a)** C1-H1-O3, **(b)** C5-S2-O1, **(c)** C5-S2-O2, **(d)** H11-N2-H12, and **(e)** N1-C12-S3.

The Electron Localization Function (ELF) serves as a powerful analytical tool for investigating the empirical concepts of electron localization, particularly the spatial confinement of electron pairs in molecular systems. This approach aligns closely with Lewis structures, offering a deeper understanding of chemical bonding by mapping electron density distributions. The ELF provides a quantitative measure of electron localization at specific points in atomic space, shedding light on bond characteristics and electronic interactions within the molecule. One of the most insightful ways to analyze ELF is through two-dimensional cross-sectional planes, which offer crucial details about bonding interactions across different molecular regions. In this study, the analysis focuses on four distinct planes: C1-H1-O3, C5-S2-O1, C5-S2-O2, and H11-N2-H12, as well as N1-C12-S3, each of which provides a unique perspective on electron distribution and bond strength. As illustrated in Figure 16, all atoms of interest are located within the same molecular plane, providing a coherent view of both localized and delocalized electron density. The selected ELF planes reveal a strongly localized electronic region (represented by the red color scale) between the S2, O1, and O2 atoms with some difference in the planes of C5-S2-O1 and C5-S2-O2, indicating a high degree of electron confinement in this bonding region. This suggests a stable electronic environment in which N2, located in the NH2 plane, plays a crucial role in molecular connectivity through the electronic delocalization effect. Generally, the plane involving the amino group (N4) exhibits an atypical electron localization pattern. The plane of C1-H1-O3 exhibits a significant H-bond formation between H1 and O3 that appears in the electronic deformation area around O3. The delocalization effect implies a dynamic charge distribution, leading to a more flexible electronic structure rather than a rigid, strongly localized bond.

##### 2.3.3.4 Molecular electrostatic potential (MEP)

**FIGURE 17:**
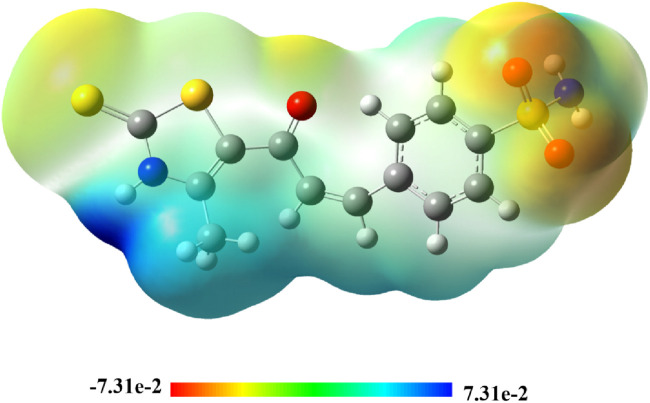
3D-colored map of the molecular electrostatic potential of compound **7**.

The Molecular Electrostatic Potential (MEP) 3D-map is a valuable computational tool used to analyze the electrostatic properties of molecules by mapping the electronegativities of atomic locations. This topological approach offers crucial insights into molecular interactions, charge distribution, and the prediction of reactive sites, thereby enhancing our understanding of molecular recognition and non-covalent interactions. Since electrostatic forces predominantly govern long-range molecular interactions, MEP plays a significant role in predicting electrophilic and nucleophilic attack sites within a given structure. To visually assess the electrostatic distribution, color-coded MEP maps were generated for compound **7**, as shown in Figure 17. These maps employ a spectrum of colors—red, orange, yellow, green, and blue—each representing different electrostatic potential values across the molecular surface. The color sequence follows the decreasing order of electrostatic potential, with orange and red indicating the most electron-rich (nucleophilic) regions. Yellow and green represent regions of moderate electronic density, typically found in neutral areas. Blue signifies electron-deficient (electrophilic) zones, which are likely to attract electron-donating species. By interpreting the MEP map, it was observed that the oxygen atoms in compound **7** exhibit the highest electron density, as highlighted by intense red zones. These regions act as strong electron-donor sites, making them highly reactive toward electrophilic attack. The remaining molecular regions, particularly the N and S atoms, exhibit a lower density of π-electrons, as represented by the yellow color scale. This suggests that these atoms are less nucleophilic compared to oxygen-rich sites. However, the area of N1 bearing H was indicated in blue, suggesting an electron donor or H-bonding formation with other molecules.

##### 2.3.3.5 Reduced density gradient/non-covalent interactions (RDG/NCI)

**FIGURE 18:**
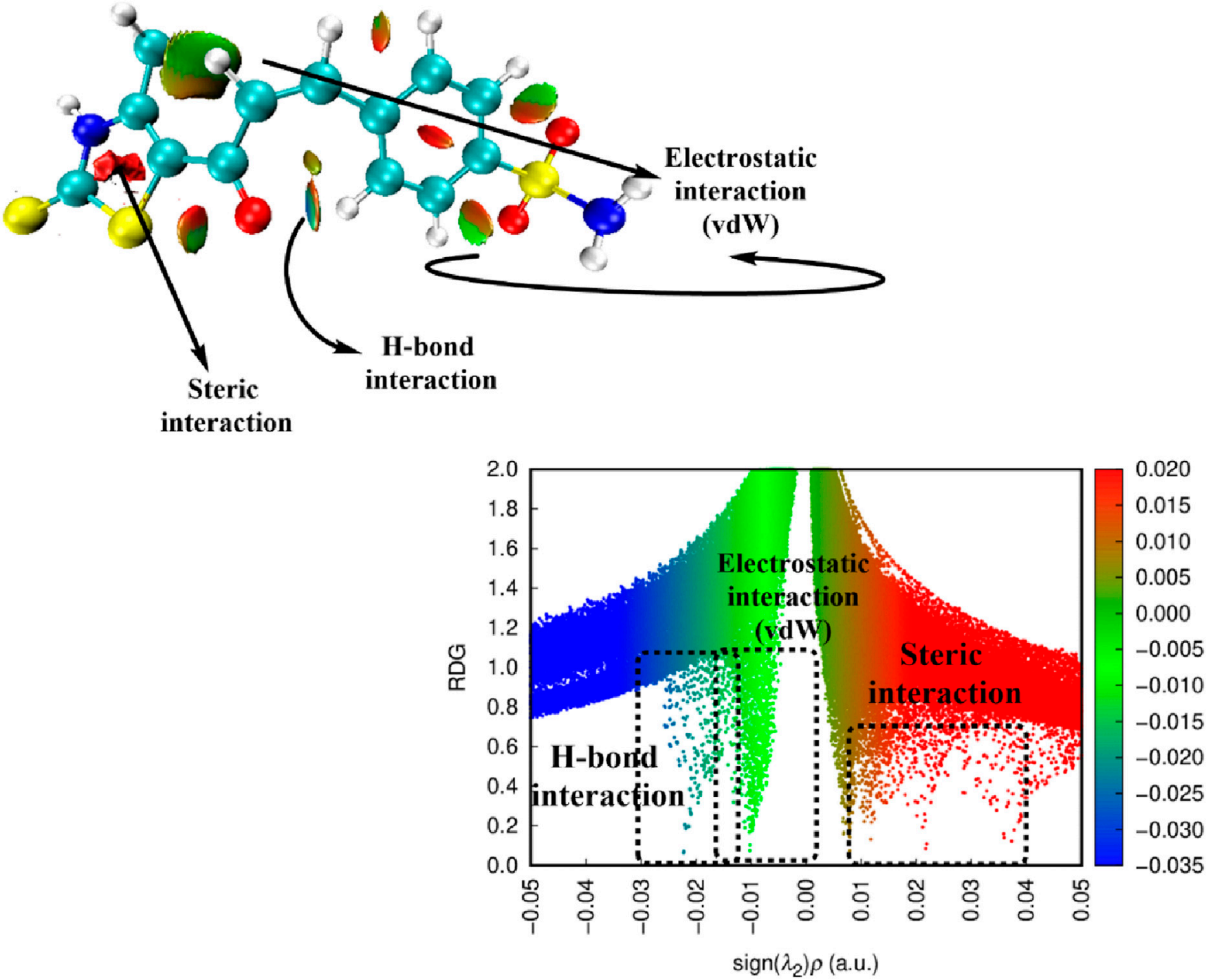
3D-NCI map and RDG plot of compound **7**.

The Reduced Density Gradient (RDG) method is a valuable computational tool for identifying and visualizing non-covalent interactions (NCIs) within a molecular system. These interactions play a crucial role in stabilizing molecular structures and influencing their chemical behavior. By analyzing the RDG function, it is possible to distinguish between different types of weak interactions, such as van der Waals (vdW) forces, hydrogen bonding, and steric repulsions. This method provides a detailed perspective on molecular stability and intra- and intermolecular forces, which are essential for predicting reactivity and interaction mechanisms**.** To effectively interpret the nature of non-covalent interactions, color-coded RDG isosurfaces were generated, as depicted in Figure 18. The different interaction types were classified based on their corresponding colors: Green regions indicate the presence of weak van der Waals (vdW) interactions, which typically contribute to molecular packing and stability. Red regions represent steric repulsions, which arise from unfavorable close contacts between atoms, often due to spatial constraints. Blue regions, if present, would indicate strong attractive interactions, such as hydrogen bonding, but were not observed in this analysis. A key parameter in this study is sign(λ_2_)ρ, which is derived by multiplying the electron density (ρ) by the sign of the second Hessian eigenvalue (λ_2_). This value serves as an indicator of interaction strength, particularly in identifying hydrogen bonding (HB) interactions within the system. From the RDG analysis, significant vdW interactions were observed, notably in the C1 and C9 regions, indicating the presence of weak attractive forces that contribute to molecular cohesion. Interestingly, hydrogen bond (H-bond) spikes were prominently detected in the RDG chart in the region between H1 and O3. The non-intense blue color is attributed to the relatively H-bearing C atom, which is predicted to be less positively charged. This reduces the sensitivity of these interactions when analyzed using the reduced density gradient approach. Although hydrogen bonding is a well-known stabilizing factor in many systems, in this case, the distance constraints prevent its strong contribution to molecular stabilization. Furthermore, steric repulsion forces were noted in regions surrounding the phenyl rings, as indicated by red zones in the RDG analysis. These repulsive interactions stem from the rigid 6- and 5-membered rings, which impose spatial constraints on molecular flexibility. However, such unfavorable steric interactions can be counterbalanced by favorable electrostatic interactions, ensuring overall molecular stability. This suggests that while steric hindrance limits certain conformational changes, the presence of stabilizing non-covalent forces, such as vdW interactions and H-bond formation, allows the structure to maintain its optimized conformation.

## 3 Conclusion

This study reports the design, synthesis, and biological evaluation of a novel thiazole–chalcone/sulfonamide hybrid (compound **7**) as a promising dual inhibitor of tubulin polymerization and carbonic anhydrase IX. The compound exhibited potent cytotoxicity against cancer cell lines, particularly HT-29 colorectal cells, with favorable selectivity over normal cells. Mechanistic investigations revealed activation of the intrinsic apoptotic pathway through modulation of p53, Bax, Bcl-2, and caspases. Molecular docking confirmed strong and specific interactions with both biological targets, while *in silico* ADMET and DFT analyses indicated suitable drug-like properties, stability, and electronic characteristics. Collectively, the data highlight compound **7** as a promising scaffold for further development as a dual-targeted anticancer agent with potential therapeutic relevance in tumors characterized by cytoskeletal dysregulation and CA IX overexpression.

## 4 Experimental

### 4.1 Chemistry

**General Details:** Refer to Appendix A (Supplementary Material).

3-Chloroacetylacetone (**2**) (Cativiela et al., 1995)**,** 1-(2-mercapto-4-methylthiazol-5-yl)ethan-1-one (**3**) (Hashem et al., 2024)**,** 4-(hydroxymethyl)benzenesulfonamide (**5**) (Procopiou et al., 2010) and 4-formylbenzenesulfonamide (**6**) (Zuo et al., 2022) were prepared according to reported procedures.

Synthesis of (*E*)-4-(3-(4-methyl-2-thioxo-2,3-dihydrothiazol-5-yl)-3-oxoprop-1-en-1-yl)benzenesulfonamide (7).

An aqueous NaOH solution (140 mg, 3.5 mmol 60%) was added dropwise to a solution of the thiazole intermediate **3** (173 mg, 1 mmol) and the aldehyde **6** (185 mg, 1 mmol) in absolute ethanol at 0°C. The reaction mixture was stirred in an ice bath for 12 h. The solvent was evaporated under reduced pressure, and the mixture was redissolved in distilled water and acidified by diluted acetic acid. The formed precipitate was filtered off, washed with distilled water, and recrystallized from acetonitrile.

Yellow powder; 0.248 g, 73% yield; mp 267–269°C; ^1^H NMR (400 MHz, DMSO-*d*_6_) δ 13.64 (s, 1H, N-*H*), 7.93 (d, *J* = 9.7 Hz, 2H, Ar-*H*), 7.83 (d, *J* = 9.2 Hz, 1H, Ar-*H*), 7.51 (d, *J* = 15.9 Hz, 1H, =C*H*), 7.41 (d, *J* = 15.9 Hz, 1H, =C*H*), 7.23 (s, 2H, SO_2_N*H*_*2*_), 2.59 (s, 3H, C*H*_*3*_); ^13^C NMR (100 MHz, DMSO-*d*_6_) δ 189.50, 180.17, 147.73, 143.96, 134.14, 131.78, 130.28, 126.41, 125.16, 123.60, 15.57. ESI-MS (*m/z*): Calcd. 340.00, found 340.41 [M]^+^. Anal. Calcd. For C_13_H_12_N_2_O_3_S_3_: C, 45.87%; H, 3.55%; N, 8.23%. Found: C, 45.99%; H, 3.71%; N, 8.04%. HPLC analysis: 97.4%.

### 4.2 Biological evaluation

#### 4.2.1 Antiproliferative assay

The cytotoxic potential of compound **7** was assessed using a propidium iodide fluorescence assay across A-549, HT-29, 786-O, MCF-7, and WI-38 cell lines. All cell lines were obtained from the Vacsera Cell Culture Library, Tissue Culture Unit, Cairo, Egypt, with ATCC certification. The assay enabled the detection of membrane-compromised cells based on the fluorescence intensity of DNA binding. Check Appendix A for additional information.

#### 4.2.2 Tubulin polymerization assay

The effect of compound **7** on tubulin polymerization was evaluated using the Tubulin Polymerization Assay Kit (Cytoskeleton Inc., United States). Fluorescence-based measurements were used to monitor its impact on microtubule dynamics. Details are presented in Appendix A (Supplementary Material).

#### 4.2.3 Evaluation of carbonic anhydrase I, II, IV, and VII inhibition

The inhibitory effect of compound **7** on carbonic anhydrase isoforms I, II, IX, and XII was evaluated using the Carbonic Anhydrase Inhibitor Screening Kit (Colorimetric, BioVision, Cat# K473-100), which measures enzymatic activity *via* substrate hydrolysis and measures absorbance at 405 nm. The assay was conducted under standard conditions as outlined by the manufacturer. Further methodological details can be found in Appendix A (Supplementary Material).

#### 4.2.4 Effect on Bax expression levels

The effect of compound **7** on pro-apoptotic Bax protein expression was evaluated using the Human Bax ELISA Kit (DRG, EIA-4487), which employs a biotin-streptavidin-HRP detection system and colorimetric measurement at 450 nm. Changes in Bax levels were quantified in treated cell lysates to assess the apoptotic response. Comprehensive assay details are available in Appendix A (Supplementary Material).

#### 4.2.5 Effect on Bcl-2 expression levels

To assess the anti-apoptotic effect of compound 7, Bcl-2 protein levels were quantified using the Zymed Bcl-2 ELISA Kit (Invitrogen, Cat. No. 99-0042), a sandwich ELISA format with streptavidin-HRP detection. Absorbance was measured at 450 nm to determine changes in Bcl-2 expression in response to treatment. A full description of the assay protocol is included in Appendix A (Supplementary Material).

#### 4.2.6 Effect on p53 expression levels

The influence of compound **7** on p53 protein levels was investigated using the Human p53 ELISA Kit (Thermo Fisher Scientific, Cat. No. BMS256), which employs a dual-antibody sandwich format with HRP-based colorimetric detection at 450 nm. The assay enabled the sensitive quantification of p53 expression following exposure to the compound. Detailed procedures and reagent preparation can be found in Appendix A (Supplementary Material).

#### 4.2.7 Effect of caspase-3 activity

The effect of compound **7** on apoptosis was further assessed by quantifying active caspase-3 levels using the Human Caspase-3 (Active) ELISA Kit (Invitrogen, Cat. No. KHO1091). This sandwich ELISA detects caspase-3 cleaved at Asp175/Ser176, enabling sensitive colorimetric measurement at 450 nm. Additional procedural details are provided in Appendix A (Supplementary Material).

#### 4.2.8 Effect on caspase-9 activity

The involvement of compound **7** in apoptosis induction was further examined by quantifying caspase-9 levels using the Human Caspase 9 ELISA Kit (Thermo Fisher Scientific, Cat. No. BMS 2025). This assay employs a sandwich ELISA format, utilizing a specific capture antibody and an HRP-conjugated detection system, with absorbance measured at 450 nm. A detailed methodology is provided in Appendix A (Supplementary Material).

### 4.3 Molecular modeling

#### 4.3.1 Molecular docking

Molecular docking of compound **7** was performed against tubulin (PDB ID: 4O2B) and carbonic anhydrase IX (PDB ID: 5FL4) using AutoDock Vina. Protein and ligand preparations, grid setup, and visualization were conducted using standard molecular modeling tools. Detailed procedures are described in Appendix A (Supplementary Material).

#### 4.3.2 ADMET predictions

The pharmacokinetic properties of compound 7 were predicted using the SwissADME web tool (http://www.swissadme.ch). The SMILES notation of the compound was input into the platform to evaluate key absorption, distribution, metabolism, and excretion (ADME) parameters. These included gastrointestinal (GI) absorption, blood-brain barrier (BBB) permeability, prediction of P-glycoprotein substrate, cytochrome P450 enzyme inhibition, and physicochemical descriptors such as lipophilicity (LogP), solubility (LogS), and topological polar surface area (TPSA). Drug-likeness was also assessed based on Lipinski’s rule of five and related filters.

#### 4.3.3 DFT calculations

The structural and electronic properties of compound **7** were investigated using density functional theory (DFT) with the B3LYP/6-311++G(d,p) level of theory. Visualization and topological analyses, including MEP mapping, RDG, NCI, and ELF, were carried out using Chemcraft, VMD, and Multiwfn software. Further computational details are provided in Appendix A (Supplementary Material).

## Data Availability

The original contributions presented in the study are publicly available. This data can be found here: https://doi.org/10.6084/m9.figshare.29369423.

## References

[B1] Abdelhakeem M. M. Morcoss M. M. Hanna D. A. Lamie P. F. (2024). Design, synthesis and *in silico* insights of novel 1,2,3-triazole benzenesulfonamide derivatives as potential carbonic anhydrase IX and XII inhibitors with promising anticancer activity. Bioorg Chem. 144, 107154. 10.1016/j.bioorg.2024.107154 38309003

[B2] Abdel-Motaal M. Aldakhili D. A. Abo Elmaaty A. Sharaky M. Mourad M. A. E. Alzahrani A. Y. A. (2024). Design and synthesis of novel tetrabromophthalimide derivatives as potential tubulin inhibitors endowed with apoptotic induction for cancer treatment. Drug Dev. Res. 85, e22197. 10.1002/ddr.22197 38751223

[B3] Abo-Ashour M. F. Eldehna W. M. Nocentini A. Ibrahim H. S. Bua S. Abdel-Aziz H. A. (2019). Novel synthesized SLC-0111 thiazole and thiadiazole analogues: determination of their carbonic anhydrase inhibitory activity and molecular modeling studies. Bioorg Chem. 87, 794–802. 10.1016/j.bioorg.2019.04.002 30978604

[B4] Ahmed N. M. Mohamed M. S. Awad S. M. Abd El-Hameed R. H. El-Tawab N. A. A. Gaballah M. S. (2024). Design, synthesis, molecular modelling and biological evaluation of novel 6-amino-5-cyano-2-thiopyrimidine derivatives as potent anticancer agents against leukemia and apoptotic inducers. J. Enzyme Inhib. Med. Chem. 39, 2304625. 10.1080/14756366.2024.2304625 38348824 PMC10866072

[B5] Ahmed R. F. Mahmoud W. R. Abdelgawad N. M. Fouad M. A. Said M. F. (2023). Exploring novel anticancer pyrazole benzenesulfonamides featuring tail approach strategy as carbonic anhydrase inhibitors. Eur. J. Med. Chem. 261, 115805. 10.1016/j.ejmech.2023.115805 37748386

[B6] Albelwi F. F. Nafie M. S. Albujuq N. R. Hourani W. Aljuhani A. Darwish K. M. (2024). Design and synthesis of chromene-1,2,3-triazole benzene sulfonamide hybrids as potent carbonic anhydrase-IX inhibitors against prostate cancer. RSC Med. Chem. 15, 2440–2461. 10.1039/d4md00302k 39026656 PMC11253856

[B50] Al-Wahaibi L. H. Elshamsy A. M. Ali T. F. S. Youssif B. G. M. Bräse S. Abdel-Aziz M. (2025) Design, synthesis, in silico studies, and apoptotic antiproliferative activity of novel thiazole-2-acetamide derivatives as tubulin polymerization inhibitors. Front. Chem. 13:1565699. 10.3389/fchem.2025.1565699 40308265 PMC12040969

[B7] Boichuk S. Galembikova A. Syuzov K. Dunaev P. Bikinieva F. Aukhadieva A. (2021). The design, synthesis, and biological activities of pyrrole-based carboxamides: the novel tubulin inhibitors targeting the colchicine-binding site. Molecules 26, 5780. 10.3390/molecules26195780 34641324 PMC8510300

[B8] Brindisi M. Kessler S. M. Kumar V. Zwergel C. (2022). Editorial: multi-target directed ligands for the treatment of cancer. Front. Oncol. 12, 980141. 10.3389/fonc.2022.980141 35992885 PMC9389393

[B9] Carta F. Supuran C. T. Scozzafava A. (2014). Sulfonamides and their isosters as carbonic anhydrase inhibitors. Future Med. Chem. 6, 1149–1165. 10.4155/fmc.14.68 25078135

[B10] Cativiela C. Serrano J. L. Zurbano M. M. (1995). Synthesis of 3-substituted pentane-2,4-diones: valuable intermediates for liquid crystals. J. Org. Chem. 60, 3074–3083. 10.1021/jo00115a023

[B11] Chiche J. Ilc K. Laferrière J. Trottier E. Dayan F. Mazure N. M. (2008). Hypoxia-inducible carbonic anhydrase IX and XII promote tumor cell growth by counteracting acidosis through the regulation of the intracellular pH. Cancer Res. 69, 358–368. 10.1158/0008-5472.CAN-08-2470 19118021

[B12] Daina A. Michielin O. Zoete V. (2017). SwissADME: a free web tool to evaluate pharmacokinetics, drug-likeness and medicinal chemistry friendliness of small molecules. Sci. Rep. 7, 42717. 10.1038/srep42717 28256516 PMC5335600

[B13] Doostmohammadi A. Jooya H. Ghorbanian K. Gohari S. Dadashpour M. (2024). Potentials and future perspectives of multi-target drugs in cancer treatment: the next generation anti-cancer agents. Cell Commun. Signal. 22, 228. 10.1186/s12964-024-01607-9 38622735 PMC11020265

[B14] Dumontet C. Jordan M. A. (2010). Microtubule-binding agents: a dynamic field of cancer therapeutics. Nat. Rev. Drug Discov. 9, 790–803. 10.1038/nrd3253 20885410 PMC3194401

[B15] El-Abd A. O. Bayomi S. M. El-Damasy A. K. Mansour B. Abdel-Aziz N. I. El-Sherbeny M. A. (2022). Synthesis and molecular docking study of new thiazole derivatives as potential tubulin polymerization inhibitors. ACS Omega 7, 33599–33613. 10.1021/acsomega.2c05077 36157722 PMC9494671

[B16] Elbadawi M. M. Eldehna W. M. Nocentini A. Abo-Ashour M. F. Elkaeed E. B. Abdelgawad M. A. (2021). Identification of *N*-phenyl-2-(phenylsulfonyl)acetamides/propanamides as new SLC-0111 analogues: synthesis and evaluation of the carbonic anhydrase inhibitory activities. Eur. J. Med. Chem. 218, 113360. 10.1016/j.ejmech.2021.113360 33773285

[B17] Elkotamy M. S. Abdelrahman M. A. Giovannuzzi S. Alkabbani M. A. Nocentini A. Supuran C. T. (2025). Development of pyrazolo[1,5-*a*]pyrimidine-grafted coumarins as selective carbonic anhydrase inhibitors and tubulin polymerization inhibitors with potent anticancer activity. Int. J. Biol. Macromol. 303, 140462. 10.1016/j.ijbiomac.2025.140462 39884639

[B18] Elshamsy A. M. Ali T. F. S. Osman M. El-Koussi N. A. (2023). Recent progress in biological activities of dihydropyrimidine derivatives: an updated mini-review. J. Adv. Biomed. Pharm. Sci. 6, 114–123. 10.21608/jabps.2023.198467.1183

[B19] Hanahan D. Weinberg R. A. (2011). Hallmarks of cancer: the next generation. Cell 144, 646–674. 10.1016/j.cell.2011.02.013 21376230

[B20] Hashem H. Hassan A. Abdelmagid W. M. Habib A. G. K. Abdel-Aal M. A. A. Elshamsy A. M. (2024). Synthesis of new thiazole-privileged chalcones as tubulin polymerization inhibitors with potential anticancer activities. Pharm. (Basel) 17, 1154. 10.3390/ph17091154 PMC11435058 39338317

[B21] Hawash M. (2022). Recent advances of tubulin inhibitors targeting the colchicine binding site for cancer therapy. Biomolecules 12, 1843. 10.3390/biom12121843 36551271 PMC9776383

[B22] Huo X.-S. Jian X.-E. Ou-Yang J. Chen L. Yang F. Lv D.-X. (2021). Discovery of highly potent tubulin polymerization inhibitors: design, synthesis, and structure-activity relationships of novel 2,7-diaryl-[1,2,4]triazolo[1,5-*a*]pyrimidines. Eur. J. Med. Chem. 220, 113449. 10.1016/j.ejmech.2021.113449 33895499

[B23] Jordan M. A. Wilson L. (2004). Microtubules as a target for anticancer drugs. Nat. Rev. Cancer 4, 253–265. 10.1038/nrc1317 15057285

[B24] Kalinin S. Malkova A. Sharonova T. Sharoyko V. Bunev A. Supuran C. T. (2021). Carbonic anhydrase IX inhibitors as candidates for combination therapy of solid tumors. Int. J. Mol. Sci. 22, 13405. 10.3390/ijms222413405 34948200 PMC8705727

[B25] Kaur K. Jaitak V. (2022). Thiazole and related heterocyclic systems as anticancer agents: a review on synthetic strategies, mechanisms of action and SAR studies. Curr. Med. Chem. 29, 4958–5009. 10.2174/0929867329666220318100019 35306982

[B26] Kavallaris M. (2010). Microtubules and resistance to tubulin-binding agents. Nat. Rev. Cancer 10, 194–204. 10.1038/nrc2803 20147901

[B27] Khokhlov A. L. Shetnev A. A. Korsakov M. K. Fedorov V. N. Tyushina A. N. Volkhin N. N. (2023). Pharmacological properties of sulfonamide derivatives, new inhibitors of carbonic anhydrase. Bull. Exp. Biol. Med. 175, 205–209. 10.1007/s10517-023-05835-w 37464193

[B28] Klika K. D. Pieve C. D. Kopka K. Smith G. Makarem A. (2021). Synthesis and application of a thiol-reactive HBED-type chelator for development of easy-to-produce Ga-radiopharmaceutical kits and imaging probes. Org. Biomol. Chem. 19, 1722–1726. 10.1039/D0OB02513E 33527964

[B29] Lembo V. Bottegoni G. (2024). Systematic investigation of dual-target-directed ligands. J. Med. Chem. 67, 10374–10385. 10.1021/acs.jmedchem.4c00838 38843874 PMC11215722

[B30] Li X. Li X. Liu F. Li S. Shi D. (2021). Rational multitargeted drug design strategy from the perspective of a medicinal chemist. J. Med. Chem. 64, 10581–10605. 10.1021/acs.jmedchem.1c00683 34313432

[B31] Lin C.-N. Tu H.-Y. Huang a-M. Hoar T.-C. Yang S.-C. Pu Y.-S. (2011). Synthesis and biological evaluation of 2′,5′-dimethoxychalcone derivatives as microtubule-targeted anticancer agents. US2011306775A1. 10.1016/j.bmc.2010.02.012 20199865

[B32] Liu W. He M. Li Y. Peng Z. Wang G. (2022). A review on synthetic chalcone derivatives as tubulin polymerisation inhibitors. J. Enzyme Inhib. Med. Chem. 37, 9–38. 10.1080/14756366.2021.1976772 34894980 PMC8667932

[B33] Lou Y. McDonald P. C. Oloumi A. Chia S. Ostlund C. Ahmadi A. (2011). Targeting tumor hypoxia: suppression of breast tumor growth and metastasis by novel carbonic anhydrase IX inhibitors. Cancer Res. 71, 3364–3376. 10.1158/0008-5472.CAN-10-4261 21415165

[B34] Makarem A. Sarvestani M. K. Klika K. D. Kopka K. (2019). A multifunctional HBED-type chelator with dual conjugation capabilities for radiopharmaceutical development. Synlett 30, 1795–1798. 10.1055/s-0039-1690194

[B35] Mboge M. Y. Mahon B. P. McKenna R. Frost S. C. (2018). Carbonic anhydrases: role in pH control and cancer. Metabolites 8, 19. 10.3390/metabo8010019 29495652 PMC5876008

[B36] McDonald P. C. Chafe S. C. Supuran C. T. Dedhar S. (2022). Cancer therapeutic targeting of hypoxia induced carbonic anhydrase IX: from bench to bedside. Cancers (Basel) 14, 3297. 10.3390/cancers14143297 35884358 PMC9322110

[B37] Mphahlele M. J. Maluleka M. M. Parbhoo N. Malindisa S. T. (2018). Synthesis, evaluation for cytotoxicity and molecular docking studies of benzo[c]furan-chalcones for potential to inhibit tubulin polymerization and/or EGFR-tyrosine kinase phosphorylation. Int. J. Mol. Sci. 19, 2552. 10.3390/ijms19092552 30154363 PMC6164331

[B38] Neri D. Supuran C. T. (2011). Interfering with pH regulation in tumours as a therapeutic strategy. Nat. Rev. Drug Discov. 10, 767–777. 10.1038/nrd3554 21921921

[B39] Procopiou P. A. Barrett V. J. Bevan N. J. Biggadike K. Box P. C. Butchers P. R. (2010). Synthesis and Structure−Activity relationships of long-acting β_2_Adrenergic receptor agonists incorporating metabolic inactivation: an antedrug approach. J. Med. Chem. 53, 4522–4530. 10.1021/jm100326d 20462258

[B40] Sgouros G. Bodei L. McDevitt M. R. Nedrow J. R. (2020). Radiopharmaceutical therapy in cancer: clinical advances and challenges. Nat. Rev. Drug Discov. 19, 589–608. 10.1038/s41573-020-0073-9 32728208 PMC7390460

[B41] Shaldam M. Eldehna W. M. Nocentini A. Elsayed Z. M. Ibrahim T. M. Salem R. (2021). Development of novel benzofuran-based SLC-0111 analogs as selective cancer-associated carbonic anhydrase isoform IX inhibitors. Eur. J. Med. Chem. 216, 113283. 10.1016/j.ejmech.2021.113283 33667848

[B42] Sultana F. Reddy Bonam S. Reddy V. G. Nayak V. L. Akunuri R. Rani Routhu S. (2018). Synthesis of benzo[d]imidazo[2,1-b]thiazole-chalcone conjugates as microtubule targeting and apoptosis inducing agents. Bioorg Chem. 76, 1–12. 10.1016/j.bioorg.2017.10.019 29102724

[B43] Supuran C. T. (2018). Carbonic anhydrase inhibitors as emerging agents for the treatment and imaging of hypoxic tumors. Expert Opin. Investig. Drugs 27, 963–970. 10.1080/13543784.2018.1548608 30426805

[B44] Trott O. Olson A. J. (2010). AutoDock Vina: improving the speed and accuracy of docking with a new scoring function, efficient optimization and multithreading. J. Comput. Chem. 31, 455–461. 10.1002/jcc.21334 19499576 PMC3041641

[B45] van Vuuren R. J. Visagie M. H. Theron A. E. Joubert A. M. (2015). Antimitotic drugs in the treatment of cancer. Cancer Chemother. Pharmacol. 76, 1101–1112. 10.1007/s00280-015-2903-8 26563258 PMC4648954

[B46] Vogelstein B. Papadopoulos N. Velculescu V. E. Zhou S. Diaz L. A. Kinzler K. W. (2013). Cancer genome landscapes. Science 339, 1546–1558. 10.1126/science.1235122 23539594 PMC3749880

[B47] Wang G. Liu W. Fan M. He M. Li Y. Peng Z. (2021). Design, synthesis and biological evaluation of novel thiazole-naphthalene derivatives as potential anticancer agents and tubulin polymerisation inhibitors. J. Enzyme Inhib. Med. Chem. 36, 1693–1701. 10.1080/14756366.2021.1958213 PMC8317958 34309466

[B48] Wang Y.-T. Huang X. Cai X.-C. Kang X.-X. Zhu H.-L. (2024). Synthesis, biological evaluation and molecular docking of thiazole hydrazone derivatives grafted with indole as novel tubulin polymerization inhibitors. J. Mol. Struct. 1301, 137343. 10.1016/j.molstruc.2023.137343

[B49] Zuo D. Do N. Hwang I. Ann J. Yu J.-W. Lee J. (2022). Design and synthesis of an N-benzyl 5-4-sulfamoylbenzylidene-2-thioxothiazolidin-4-one scaffold as a novel NLRP3 inflammasome inhibitor. Bioorg Med. Chem. Lett. 65, 128693. 10.1016/j.bmcl.2022.128693 35314328

