# Supplementary material for: A novel thiazole-sulfonamide hybrid molecule as a promising dual tubulin/carbonic anhydrase IX inhibitor with anticancer activity
Source: Front Chem. 2025 Jun 26;13:1606848. doi: 10.3389/fchem.2025.1606848 (PMC12260535; doi:10.3389/fchem.2025.1606848)
Supplement: Supplementary file 1 [file DataSheet1.docx]

**Supporting information**

**A Novel Thiazole-Sulfonamide Hybrid Molecule as a promising Dual Tubulin/Carbonic Anhydrase IX Inhibitor with Anticancer Activity**

Hussam Elddin Nabeih Khasawneh^1^, Elryah I. Ali^2^, Ranya Mohammed Elmagzoub^*2^, Raed Fanoukh Aboqader Al-Aouadi^3^, Wesam Taher Almagharbeh^4^, Ghallab Alotaibi^5^, Stefan Bräse^*6^, Abdullah Alkhammash^5^

^1^ Chemical Engineering Department, Al-Huson University College, Al-Balqa’ Applied University, Al-Salt P.O. Box 19117, Jordan

^2^ Department of Medical Laboratory Technology, College of Applied Medical Sciences, Northern Border University, Arar, Saudi Arabia

^3^ College of Medicine ,Al-Ayen Iraqi University,AUIQ, An Nasiriyah ,Iraq

^4^ Medical and Surgical Nursing Department, Faculty of Nursing, University of Tabuk, Tabuk 71491, Saudi Arabia

^5^ Department of Pharmacology, College of Pharmacy, Al-Dawadmi Campus, Shaqra University, Shaqra, 11961, Saudi Arabia

^6^ Institute of Biological and Chemical Systems, Functional Molecular Systems (IBCS-FMS), Karlsruhe Institute of Technology (KIT), Kaiserstrasse 12, 76131 Karlsruhe, Germany

*To whom correspondence should be addressed

**
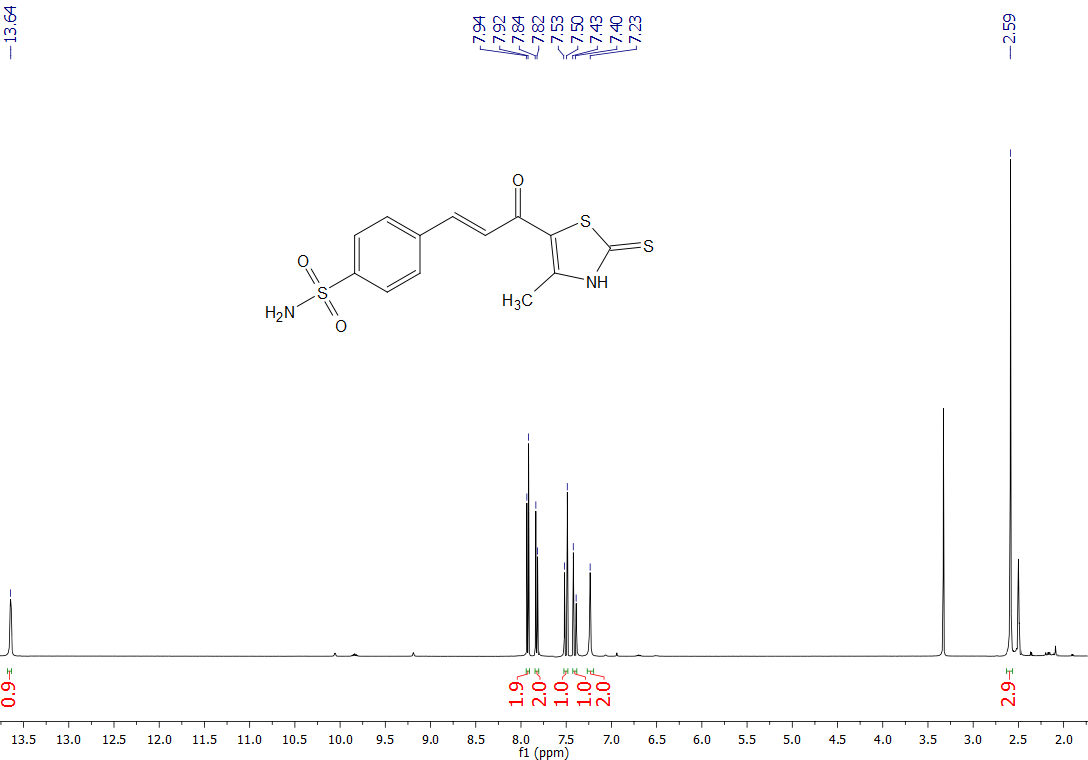
**

**Figure S1.** ^1^H-NMR spectrum of the target compound (400 MHz, DMSO-*d*_6_)


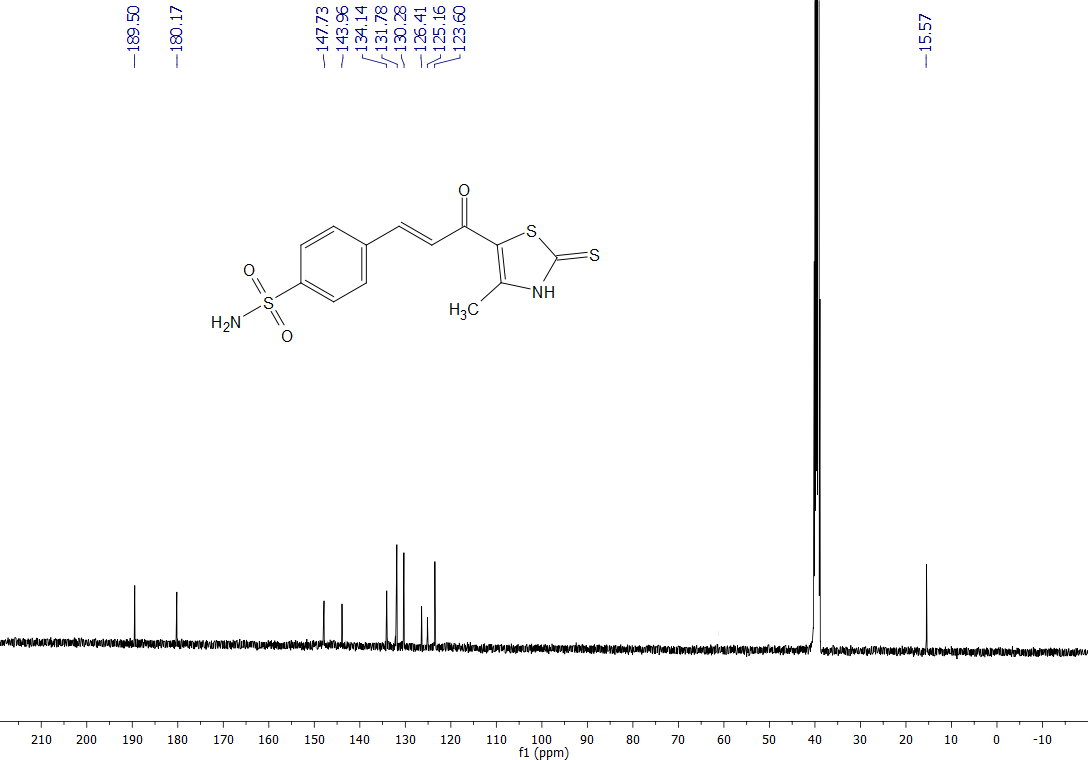


**Figure S2**. ^13^ C-NMR spectrum of the target compound (100 MHz, DMSO-*d*_6_)

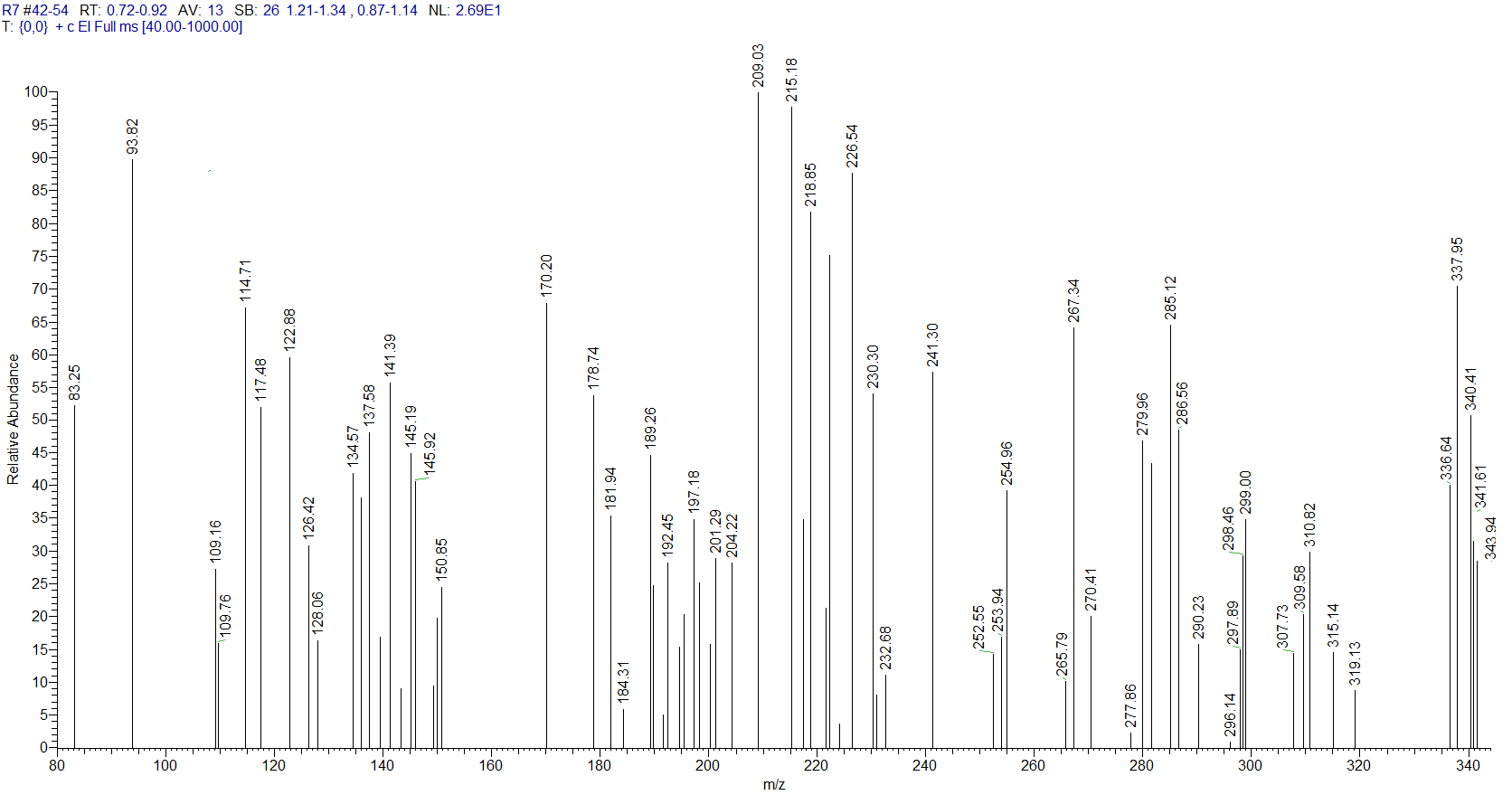


**Figure S3.** Mass spectrum of the target compound


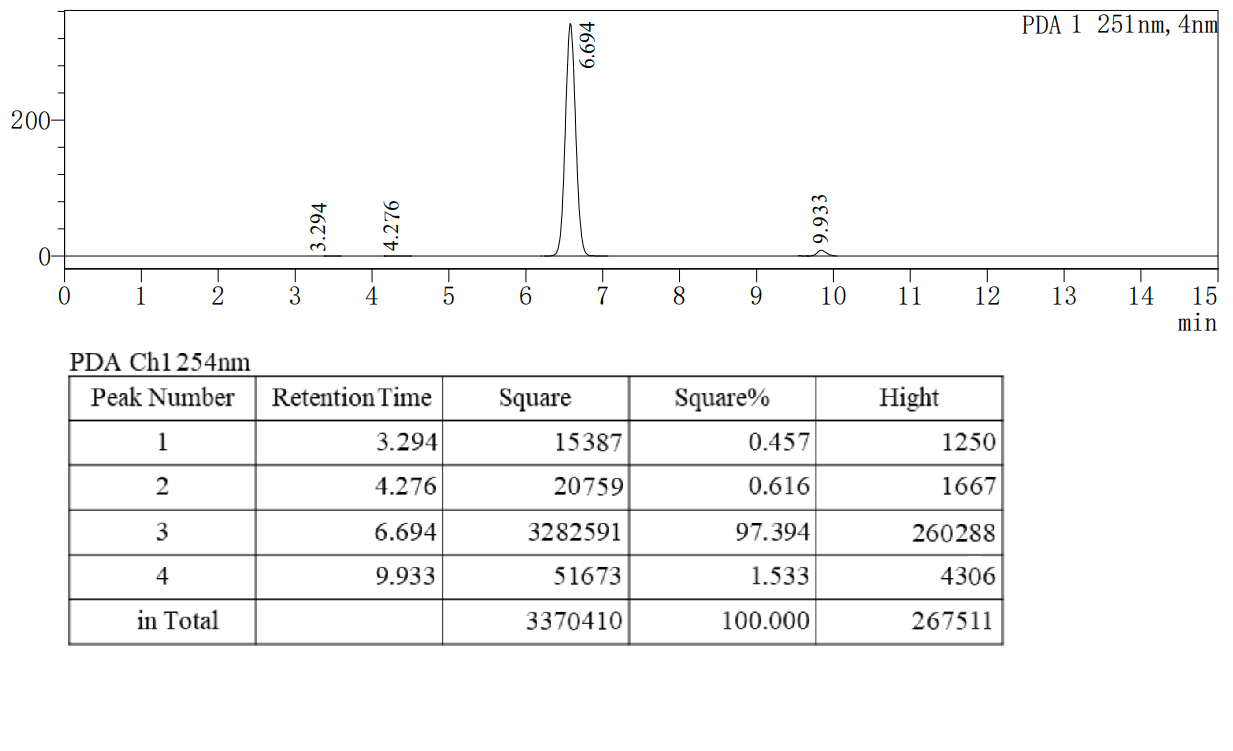


**Figure S4.** HPLC analysis of the target compound

**Appendix A**

**4. Experimental**

**4.1. Chemistry**

***General Information***

All reagents and solvents were of general purpose or analytical grade and purchased from Sigma Aldrich Ltd, Fisher Scientific, Fluka and Acros. ^1^H- and ^13^C-NMR spectra were recorded with a Bruker Avance III spectrometer operating at 400, 100 MHz respectively, with Me_4_Si as internal standard and DMSO-*d_6_* as a solvent. Elemental analysis was performed by the regional center for mycology and biotechnology (Cairo, Egypt). TLC was carried out on precoated silica plates (Keisel gel 60 F254, BDH) using Hexane: Ethyl acetate, 1 : 2, v/v. Compounds were visualized by illumination under UV light (254 nm). Melting points were determined on an electrothermal instrument and are uncorrected. All solvents were dried prior to use and stored over 4 Å molecular sieves, under nitrogen. All the compounds were ≥ 95% pure.

**Synthesis of intermediate 3**

A solution of sulfuryl chloride (1.62 g, 12 mmol) in toluene was added dropwise to a solution of acetylacetone (1 g, 10 mmol) in toluene then the reaction mixture was allowed to stir at room temperature for 16 h. The mixture was then washed with 10% aqueous sodium carbonate solution several times, dried and evaporated to yield 3-chloroacetyl acetone as a brown oil. This oil was redissolved in absolute ethanol and to it was added dropwise a previously mixed solution of ammonia and carbon disulfide in ethanol and the resulting mixture was refluxed for 6 h. Then the solvent was evaporated under reduced pressure and the residue was washed extensively with distilled water and diethyl ether, dried and recrystallized from acetonitrile.

Yellow crystals; 1.06 g, 61 % yield; mp 148-150 ̊C (Lit. mp 151–152 °C[1])

**Synthesis of intermediate 5**

A solution of BH_3_ in THF (8.6 ml, 90 mmol) was added dropwise to a solution of 4-sulfamoylbenzoic acid (0.603 g, 3 mmol) in THF at 0^°^C then the resulting solution was stirred at room temperature for 19 h. Then 2M HCl was added dropwise in ice bath and stirred for 3 h at RT. The mixture was extracted using ethyl acetate, dried and evaporated. The formed residue was recrystallized from aqueous ethanol.

White crystals; 0.438 g, 78 % yield; mp 117-119 ̊C (Lit. mp 118–120 °C[2])

**Synthesis of intermediate 6**

Pyridinium chlorochromate (0.992 g, 4.6 mmol) was added portionwise to a suspension of compound 5 (0.430 g, 2.3 mmol) in dichloromethane and the reaction mixture was allowed to stir at reflux for 6 h. The solvent was evaporated in vacuo and the residue was redissolved in acetone. The mixture was filtered then the filterate was evaporated and the formed residue was recrystallized from boiling water.

White powder; 0.234 g, 55 % yield; mp 113-114 ̊C (Lit. mp 117–118 °C[3])

**4.2. Biological evaluation**

**2.1. Antiproliferative Assay**

The cytotoxic effect of compound 7 was evaluated against four human cancer cell lines: A549 (lung carcinoma), HT-29 (colon adenocarcinoma), 786-O (renal carcinoma), and MCF-7 (breast adenocarcinoma), as well as the normal human lung fibroblast cell line WI-38. Cells were cultured in their respective recommended media supplemented with 10% fetal bovine serum and 1% penicillin-streptomycin and maintained at 37 °C in a humidified atmosphere containing 5% CO₂. Cell viability was assessed using the MTT assay, following the protocol provided with the *In Vitro* Toxicology Assay Kit, MTT Based (Sigma-Aldrich, TOX-1). Briefly, cells were seeded in 96-well plates at a density of 5 × 10³ cells/well and allowed to adhere overnight. The following day, cells were treated with varying concentrations of compound 7 and incubated for 48 h. Subsequently, 10 µL of reconstituted MTT solution (5 mg/mL in serum-free, phenol red-free medium) was added to each well and incubated for 3 h at 37 °C. The resulting formazan crystals were dissolved by adding 100 µL of the MTT Solubilization Solution, and the plates were gently shaken to ensure complete solubilization. Absorbance was recorded at 570 nm with a reference wavelength of 690 nm using a microplate reader. Cell viability was expressed as a percentage relative to untreated control cells. All experiments were conducted in triplicate, and IC₅₀ values were determined using linear regression analysis.

**2.2. Tubulin polymerization assay**

The effect of compound 7 on tubulin polymerization was evaluated using the Tubulin Polymerization Assay Kit (Fluorescence-based, Cytoskeleton, Inc., Cat. No. BK011P), according to the manufacturer’s protocol. This assay monitors the assembly of purified porcine brain tubulin into microtubules by measuring fluorescence enhancement due to incorporation of a fluorescent reporter into the growing microtubules. Briefly, compound 7 was prepared as a 10× stock solution in DMSO and diluted to desired concentrations in assay buffer. Each well of a pre-warmed 96-well black flat-bottom plate received 5 µL of compound 7 solution or control buffer. The reaction was initiated by adding 50 µL of tubulin reaction mix (2 mg/mL tubulin in 80 mM PIPES, pH 6.9, 2.0 mM MgCl₂, 0.5 mM EGTA, 1.0 mM GTP, and 15% glycerol). The plate was immediately transferred to a temperature-controlled microplate reader pre-equilibrated at 37 °C. Polymerization was monitored kinetically by measuring fluorescence at 360 nm excitation and 420 nm emission every minute for 60 minutes. Control wells included vehicle control and paclitaxel (as a polymerization enhancer) or vinblastine (as a polymerization inhibitor). Changes in the polymerization kinetics, including the nucleation lag phase and V_max_, were used to assess the effect of compound 7. IC_50_ values were calculated using linear regression analysis from the obtained fluorescence curves.

**2.3. Evaluation of Carbonic anhydrase I, II, IV, and VII inhibition**

The inhibitory activity of compound 7 against carbonic anhydrase (CA) isoforms I, II, IX, and XII was assessed using the Carbonic Anhydrase Inhibitor Screening Kit (Colorimetric, BioVision, Cat. No. K473-100), following the manufacturer's protocol. The assay is based on the esterase activity of active CA enzymes on a chromogenic substrate, which produces a measurable absorbance at 405 nm. Recombinant human CA isoforms I, II, IX, and XII were incubated with varying concentrations of compound 7 in 96-well plates. Each well contained 80 µL of CA assay buffer, 5 µL of reconstituted CA enzyme, and 10 µL of compound 7 solution (dissolved in DMSO), followed by a 10-minute incubation at room temperature. The reaction was initiated by adding 5 µL of CA substrate, and absorbance was measured at 405 nm in kinetic mode for 60 minutes using a microplate reader. The enzymatic activity in the presence of compound 7 was compared to enzyme control wells (without inhibitor). The relative activity and percentage inhibition were calculated. IC₅₀ values were determined using linear regression analysis from dose–response curves.

**2.4. Effect on *BAX* expression levels**

The level of *BAX* protein expression following treatment with compound 7 was determined using the Human *BAX* ELISA Kit (DRG International, Inc., Cat. No. EIA-4487) according to the manufacturer's instructions. This sandwich-based immunoassay utilizes a monoclonal antibody specific for human *BAX*-α to capture and quantify the protein in cell lysates. Cells were lysed using the provided Cell Lysis Buffer, freshly supplemented with phenylmethylsulfonyl fluoride (PMSF, 1 mM) and protease inhibitor cocktail (PIC, 0.5 µL/mL). Lysates were incubated on ice, vortexed, and centrifuged at 16,000 rpm for 15 minutes at 4 °C. The supernatants were collected and diluted with the kit's Assay Buffer. A standard curve was generated using *BAX*-α standards with concentrations of 62.5, 125, 250, 500, 1000, and 2000 ng/mL. The corresponding net absorbance values (after subtracting the average blank OD of 0.023) were 0.110, 0.192, 0.338, 0.781, 1.443, and 2.496, respectively. Absorbance was measured at 450 nm with a reference wavelength of 570–590 nm. *BAX* concentrations in the samples were calculated using linear regression analysis based on the standard curve obtained. The assay provided a sensitive and quantitative measure of *BAX* modulation in response to compound 7.

**2.5. Effect on *Bcl-2* expression levels**

The *Bcl-2* protein levels following treatment with compound 7 were quantified using the Zymed® *Bcl-2* ELISA Kit (Cat. No. 99-0042), following the manufacturer’s instructions. The assay is based on a sandwich ELISA format, where human *Bcl-2* is captured by a monoclonal antibody coated on a microwell plate and detected using a biotin-conjugated anti-*Bcl-2* antibody followed by Streptavidin-HRP and TMB substrate. Cells were lysed using the supplied Lysis Buffer at a concentration of 5 × 10⁶ cells/mL and incubated for 1 hour at room temperature with gentle shaking. Lysates were clarified by centrifugation at 1000 × g for 15 minutes, and the supernatants were collected for immediate use or stored at –80 °C. For the assay, 20 µL of sample was added to wells along with 80 µL of Sample Diluent, followed by 50 µL of diluted biotin-conjugate. After a 2-hour incubation at room temperature, plates were washed and incubated with diluted Streptavidin-HRP for 1 hour. TMB substrate was then added, and color development was allowed for 15 minutes before stopping the reaction with phosphoric acid. Absorbance was measured at 450 nm with optional correction between 610–650 nm. A standard curve was constructed using *Bcl-2* standards at concentrations of 1, 2, 4, 8, 16, and 32 ng/mL. The corresponding absorbance values ranged from approximately 0.121 to 2.461, with a background (blank) absorbance of 0.023. Net absorbance values were calculated by subtracting the blank from each standard, and the resulting data were used to construct a calibration curve. *Bcl-2* concentrations in unknown samples were then interpolated using linear regression analysis based on the standard curve.

**2.6. Effect on *p53* expression levels**

The *p53* protein concentration following treatment with compound 7 was determined using the Human *p53* ELISA Kit (Thermo Fisher Scientific, Cat. No. BMS256), following the manufacturer's protocol. The assay utilizes a sandwich ELISA format in which human *p53* is captured by an immobilized monoclonal antibody and detected using a biotin-conjugated secondary antibody, followed by Streptavidin-HRP and TMB substrate. After color development, the reaction is stopped and absorbance is measured at 450 nm. Samples were diluted 1:2 in Sample Diluent and loaded into the wells, followed by addition of the biotin-conjugated antibody. Plates were incubated at room temperature for 2 hours, washed, and incubated for 1 hour with diluted Streptavidin-HRP. Following additional washing steps, TMB substrate was added, and the color was developed for approximately 10 minutes. The reaction was stopped with phosphoric acid, and absorbance was immediately recorded at 450 nm with reference at 620 nm. A standard curve was generated using serial dilutions of reconstituted human *p53* standard, yielding final concentrations of 125, 250, 500, 1000, 2000, 4000, and 8000 pg/mL. The corresponding absorbance values ranged from 0.193 to 2.594, with a blank reading of 0.136. The average blank value was subtracted from all readings, and a calibration curve was plotted. The concentration of *p53* in the treated samples was determined by linear regression analysis of the standard curve. All results were multiplied by the dilution factor to obtain the final values.

**2.7. Effect of *caspase-3* activity**

The level of active *caspase-3* protein following treatment with compound 7 was quantified using the Human Active *Caspase-3* ELISA Kit (Invitrogen, Cat. No. KHO1091) according to the manufacturer’s instructions. This assay is based on a solid-phase sandwich ELISA principle, where an anti-human *caspase-3* monoclonal antibody is pre-coated onto a microplate. Cell lysates or standards are captured by this antibody, followed by the addition of a rabbit polyclonal detection antibody specific for active *caspase-3* cleaved at Asp175/Ser176. Detection is then performed using HRP-conjugated anti-rabbit IgG and TMB substrate. Cell lysates were prepared in protease inhibitor-supplemented cell extraction buffer and clarified by centrifugation. Samples were diluted in Standard Diluent Buffer and added to the wells along with a standard curve of human active *caspase-3*. The standard concentrations included 39, 78, 156, 313, 625, 1250, and 2500 ng/mL, which yielded optical density (OD) readings ranging from 0.192 to 2.733. A blank reading of 0.018 was subtracted from each standard OD, and the resulting data were used to generate a standard curve. Following incubation and sequential wash steps, the detection antibody, HRP conjugate, and TMB substrate were added. After color development, the reaction was stopped with acid, and absorbance was read at 450 nm. The concentration of active *caspase-3* in treated samples was determined using linear regression analysis of the standard curve. All measurements were adjusted by the dilution factor.

**2.8. Effect of *caspase-9* activity**

The concentration of human *caspase-9* protein following treatment with compound 7 was quantified using the Human *Caspase-9* ELISA Kit (Thermo Fisher Scientific, Cat. No. BMS2025), following the manufacturer’s protocol. This solid-phase sandwich ELISA employs a monoclonal antibody pre-coated on a 96-well microplate to capture *caspase-9* from samples. A rabbit polyclonal detection antibody specific to human *caspase-9* is subsequently added, followed by an HRP-conjugated anti-rabbit IgG and TMB substrate for colorimetric detection. The intensity of the yellow product formed after stopping the reaction with phosphoric acid is proportional to the amount of *caspase-9* present and is measured at 450 nm. Cell lysates were prepared in 1× lysis buffer from the kit, using approximately 5 × 10⁶ cells/mL, followed by 60 minutes of incubation at room temperature with gentle agitation. Lysates were centrifuged at 1,000 × g for 15 minutes, and the clear supernatants were collected and either analyzed immediately or stored at –80 °C. Samples were diluted 1:2 in Sample Diluent and added to the microplate along with a standard curve prepared via 1:2 serial dilutions of the reconstituted standard. Standard concentrations of 1.6, 3.1, 6.3, 12.5, 25, 50, and 100 ng/mL yielded OD values of 0.140, 0.167, 0.213, 0.239, 0.501, 0.799, and 1.639 respectively, with a blank reading of 0.042. Net OD values were calculated by subtracting the blank absorbance, and the standard curve was constructed accordingly. The concentration of *caspase-9* in each sample was determined using linear regression analysis, and final values were adjusted by the dilution factor.

**3. Molecular Modeling**

**3.1. Molecular docking**

The crystal structures of tubulin-colchicine complex (PDB code: 4O2B) and human carbonic anhydrase IX (PDB code: 5FL4) were downloaded from the Protein Data Bank. Structure of compound 7 was drawn and optimized using MarvinSketch and Avogadro molecular editors. The proteins were prepared using autodock tools where the co-crystallized water molecules and colchicine were removed then kollman charges and polar hydrogens were added. The grid coordinates for tubulin were set to 15.951x66.804x43.33 for x, y and z axes, respectively with grid dimensions of 80x80x80. Autodock vina was used for molecular docking and the best docking poses were visualized using Discovery Studio Visualizer.

**3.2. ADMET predictions**

The pharmacokinetic properties of compound 7 were predicted using the SwissADME web tool (<http://www.swissadme.ch>). The SMILES notation of the compound was input into the platform to evaluate key absorption, distribution, metabolism, and excretion (ADME) parameters. These included gastrointestinal (GI) absorption, blood-brain barrier (BBB) permeability, P-glycoprotein substrate prediction, cytochrome P450 enzyme inhibition, and physicochemical descriptors such as lipophilicity (LogP), solubility (LogS), and topological polar surface area (TPSA). Drug-likeness was also assessed based on *Lipinski’s rule of five* and related filters.

**3.3. DFT calculations**

Compound 7 was analyzed computationally through density functional theory (DFT) to achieve complete optimization of its structural and electronic properties, utilizing the hybrid B3LYP functional. The calculations were done with the 6-311++G(d,p) basis set via Gaussian 09 software. Molecular electrostatic potential (MEP) mapping was performed to pinpoint the optimized molecule's key electrophilic and nucleophilic regions. The compound’s most stable structure and electronic excitation states were visualized using Chemcraft and VMD software. Furthermore, additional topological analyses, including reduced density gradient (RDG) and non-covalent interaction (NCI) studies, were performed using Multiwfn software to explore intramolecular interactions. Electron localization function (ELF) analysis was also conducted to provide deeper insight into bonding characteristics and electron distribution within the heterocyclic framework.

**References**

1. D’Amico, J.J. Thiazolethiols and Their Derivatives. *J. Am. Chem. Soc.* **1953**, *75*, 102–104, doi:10.1021/ja01097a029.

2. Tashika, Y.; Nitta, Y.; Yomoda, J.; Oya, H. Synthesis of p -Sulfamylbenzyl Alcohol. *Yakugaku Zasshi-journal of The Pharmaceutical Society of Japan* **1952**, *72*, 398–400, doi:10.1248/YAKUSHI1947.72.3_398.

3. Van Es, T.; Staskun, B. ALDEHYDES FROM AROMATIC NITRILES: 4-FORMYLBENZENESULFONAMIDE. *Org. Synth.* **1971**, *51*, 20, doi:10.15227/orgsyn.051.0020.
